# Strategies for the production of cell wall‐deconstructing enzymes in lignocellulosic biomass and their utilization for biofuel production

**DOI:** 10.1111/pbi.12505

**Published:** 2015-12-02

**Authors:** Sang‐Hyuck Park, Rebecca Garlock Ong, Mariam Sticklen

**Affiliations:** ^1^ Department of Plant, Soil and Microbial SciencesMichigan State UniversityEast LansingMIUSA; ^2^ Department of Chemical Engineering and Materials ScienceDOE Great Lakes Bioenergy Research CenterMichigan State UniversityLansingMIUSA; ^3^ Department of Chemical EngineeringMichigan Technological UniversityHoughtonMIUSA

**Keywords:** cell wall‐deconstructing enzymes, lignocellulosic biomass, biofuels, promoter, plant subcellular compartments, signal peptides

## Abstract

Microbial cell wall‐deconstructing enzymes are widely used in the food, wine, pulp and paper, textile, and detergent industries and will be heavily utilized by cellulosic biorefineries in the production of fuels and chemicals. Due to their ability to use freely available solar energy, genetically engineered bioenergy crops provide an attractive alternative to microbial bioreactors for the production of cell wall‐deconstructing enzymes. This review article summarizes the efforts made within the last decade on the production of cell wall‐deconstructing enzymes *in planta* for use in the deconstruction of lignocellulosic biomass. A number of strategies have been employed to increase enzyme yields and limit negative impacts on plant growth and development including targeting heterologous enzymes into specific subcellular compartments using signal peptides, using tissue‐specific or inducible promoters to limit the expression of enzymes to certain portions of the plant or certain times, and fusion of amplification sequences upstream of the coding region to enhance expression. We also summarize methods that have been used to access and maintain activity of plant‐generated enzymes when used in conjunction with thermochemical pretreatments for the production of lignocellulosic biofuels.

## Introduction

Plant lignocellulosic biomass is mainly comprised of polymeric sugars (cellulose, hemicellulose and pectin), and polyphenolics (lignin) that are found within the plant cell wall (Hu *et al*., 2012; Li *et al*., 2014). During the biochemical conversion of lignocellulosic biomass to fuels, cell wall‐deconstructing enzymes are used to convert plant cell wall polysaccharides into fermentable sugars. In addition to their use in the bioethanol industry, these enzymes are also widely used in agricultural waste bioremediation, as well as by the pulp and paper, cosmetics and textiles industries. In the pulp and paper industry, enzyme treatments are considered more cost‐effective compared with mechanical processes, resulting in up to 20%–40% energy savings (Kuhad *et al*., 2011). However, the costs associated with the production of microbial enzymes for use by the biofuel industry are expected to be high. These expected costs vary widely, ranging from ~$0.34 to $1.47 per gallon of cellulosic ethanol produced, with enzyme companies stating ~$0.50/gallon of ethanol (Hong *et al*., 2013; Humbird *et al*., 2011; Klein‐Marcuschamer *et al*., 2012). Even at the low end, the enzyme cost is expected to account for ~15% of the minimum ethanol selling price and 25% of the total biorefinery processing costs (Humbird *et al*., 2011).

Producing enzymes in plants is one strategy to reduce the production and processing costs of cell wall‐deconstructing enzymes. It has been estimated that the cost for producing heterologous proteins in plants is at least 10–50 fold less compared with their production in microbes (Giddings *et al*., 2000). Currently, the USA is capable of generating between 500 million and 1.5 billion tons of lignocellulosic biomass every year, with 100–800 million tons of bioenergy crops and 150–400 million tons of agricultural residues (Perlack and Stokes, 2011). Should 0.5% of the herbaceous plants be directed for production of cell wall‐deconstructing enzymes (Egelkrout *et al*., 2012), this could generate as much as 6 million tons of cell wall‐deconstructing enzymes every year.

This review article summarizes the efforts made within the last decade on the production of cell wall‐deconstructing enzymes *in planta*, focusing on current strategies to increase the yield of enzymes in plant vegetative biomass. These strategies include targeting the heterologous enzymes into specific subcellular compartments via signal peptides, using tissue‐specific or inducible promoters to limit the interference of enzymes with cellular function, and fusion of amplification sequences upstream of the coding region to enhance expression. We also address a number of issues that can occur during expression of enzymes in plant materials. Finally, we summarize various studies on the use of heterologously produced enzymes in conjunction with thermochemical pretreatment methods to increase the yields of fermentable sugars generated from lignocellulosic biomass.

## Subcellular targeting of heterologous enzymes

**Figure 1:**
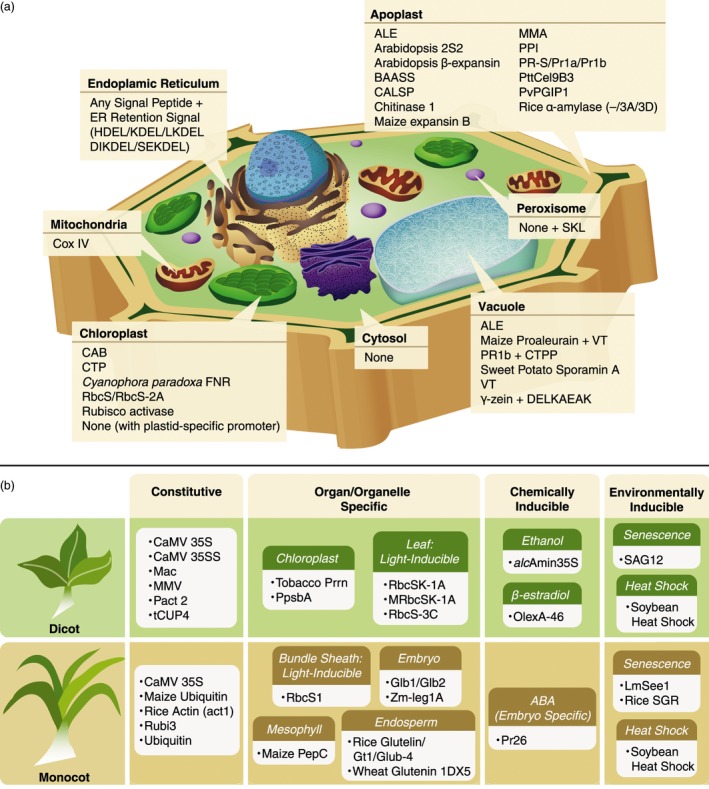
(a) Signal peptides used for accumulation of cell wall‐degrading enzymes in plant subcellular compartments; (b) promoters used for expression of cell wall‐degrading enzymes in monocots and dicots. *alc*Amin35S, alcohol‐inducible promoter based on CaMV 35S; ALE, barley aleurain vacuole‐targeting signal; BAASS, Barley α‐amylase signal sequence; CAB, Chlorophyll *a‐/b*‐binding protein; CALSP, tobacco calreticulin signal peptide; CaMV35SS, double CaMV 35S promoter; Cox IV, Yeast cytochrome c oxidase subunit; CTP, artificial dicot chloroplast targeting sequence; CTPP, C‐terminal propeptide tobacco chitinase vacuolar sorting signal; DELKAEAK, vacuole sorting determinant; FNR, ferredoxin‐NADP ^+^‐oxidoreductase; Glb1, Maize globulin1; Glb2, globulin2; Glub‐4, rice glutelin B‐4 gene; Gt1, rice glutelin Gt1 promoter; (SE/DI/L)KDEL/HDEL, endoplasmic reticulum retention signal; LmSee1, Lolium multiflorum senescence‐enhanced gene promoter; Mac, hybrid of Ti plasmid mannopine synthetase promoter and cauliflower mosaic virus 35S promoter enhancer; MMA, leader peptide derived from murine monoclonal antibody mAb24; MMV, Mirabilis mosaic virus promoter; MRbcSK‐1A, three alfalfa RbcS promoters (RbcSK‐1A) without negative regulatory region; OlexA‐46, β‐estradiol‐inducible promoter; Pact2, Arabidopsis actin 2 promoter; PepC, phosphoenolpyruvate carboxylase; PpsbA, PSII protein D1 promoter; PPI, Potato protease inhibitor II; Prrn, Tobacco 16S ribosomal ERNA promoter; PR‐S/PR1a/PR1b, pathogenesis‐related proteins; PvPGIP1, Phaseolus vulgaris polygalacturonase‐inhibiting protein; RbcS, Rubisco small subunit; Rice SGR, Rice Stay Green gene; Rubi3, rice ubiquitin promoter; SAG12, Arabidopsis senescence‐inducible promoter; SKL, peroxisome‐targeting C‐terminal sequence; VT, vacuole‐targeting signal peptide; Zm‐leg1A, maize legumin promoter.

Plant cells harbour several functionally specialized subcellular compartments including the endoplasmic reticulum (ER), chloroplast, mitochondria, Golgi, peroxisome and vacuole. It is possible to target recombinant cell wall‐deconstructing enzymes for accumulation in specific subcellular compartments using a signal peptide, a short, transient peptide located at the N‐terminus of a newly synthesized protein that directs the protein for secretion or to a specific organelle. Once delivered to its final destination, the signal peptide is usually cleaved off of the enzyme by a signal peptidase. Such targeting is valuable for a number of reasons. The organelles can be targeted based on their compatibility with the physical properties of the enzymes (e.g. pH stability). Also enzymes can be sequestered inside organelles until plant maturation in order to protect the plant cell walls from premature degradation. Many signal peptides have been identified that target different subcellular compartments, a number of which have been used to direct storage of heterologously produced microbial enzymes (Figure 1a).

### Targeting the cytosol for enzyme accumulation

Targeting the cytosol for enzyme expression simply involves the removal of any signalling peptide from the gene construct (Ziegelhoffer *et al*., 1999). In one of the first studies on heterologous expression of enzymes *in planta*, a cellobiohydrolase (E2/Cel6A) and an endoglucanase (E3/Cel6B) from *Thermobifida fusca* (formerly *Thermomonospora fusca*) were targeted into the cytosol of tobacco and alfalfa, albeit at extremely low levels (Ziegelhoffer *et al*., 1999). Since then, a large number of scientific teams have examined the feasibility of producing a number of different enzymes in the cytosol (Table 1), with levels ranging from 0% to 50% TSP (Table S1). In spite of this, the cytosol may not be the best location for storing heterologous enzymes due to the presence of high levels of proteases and the lack of co‐ and post‐translational modifications that are required for proper enzymatic functioning (Egelkrout *et al*., 2012). However, it is possible to use protease inhibitors to protect newly synthesized enzymes against degradation either when produced in the cytosol, or when they are migrating through the cytosol towards their targeted compartments (Goulet *et al*., 2010, 2012).

**Table 1:** Summary of subcellular targeting of cell wall‐deconstructing enzymes since 2005. A full version of the table is available in the supplemental information (Table S1)

Targeting compartment	Signal peptide/ termination sequence	Host plants and promoters	Heterologous enzyme	References
Cytosol	–	Arabidopsis: RbcSK‐1A, CaMV 35S Duckweed: CaMV 35S Potato: CaMV 35SS Rice: CaMV 35S, Maize ubiquitin, Rice actin act1, Rice SGR, Ubiquitin Rice Seeds: Gt1 Tobacco: Mac, RbcSK‐1A, MRbcSK‐1A + aps, Pact2, CaMV 35S, CaMV 35SS, MMV	Endo‐1,4‐β‐glucanase (3.2.1.4) Cellulose 1,4‐β‐cellobiosidase^NR^ (3.2.1.91) 1,4‐β‐glucosidase (3.2.1.21) Endo‐1,4‐β‐xylanase (3.2.1.8) Polygalacturonase (3.2.1.15) 4‐O‐methyl‐glucuronoyl methylesterase (3.2.1.73) Endo‐1,4‐β‐xylanase (3.2.1.8) Feruloyl esterase (3.1.1.73)^a^ Laccase (1.10.3.2) + CBM *Chimeric*: Endo‐1,4‐β‐xylanase (3.2.1.8) Feruloyl esterase (3.1.1.73) α‐arabinofuranosidase^a^ (3.2.1.55)	Bae *et al*. (2006, 2008), Chatterjee *et al*. (2010), Dai *et al*. (2005), Fan and Yuan (2010), Furukawa *et al*. (2014, 2013), Hahn *et al*. (2014), Jiang *et al*. (2011), Jung *et al*. (2010, 2013), Kimura *et al*. (2010), Mahadevan *et al*. (2011), Nigorikawa *et al*. (2012), Pereira *et al*. (2014), Sun *et al*. (2007), Tsai *et al*. (2012), Weng *et al*. (2013), Yang *et al*. (2007) and Zhang *et al*. (2012)
Apoplast	Mutated ALE; Mutated ALE/Frameshift KDEL	Tall Fescue: Rice Actin 1, Soya bean Heat Shock, LmSee1	Feruloyl esterase (3.1.1.73)	Buanafina *et al*. (2010)
	Arabidopsis 2S2	Tobacco: MMV	Endo‐1,4‐β‐xylanase (3.2.1.8) Feruloyl esterase (3.1.1.73)^a^	Chatterjee *et al*. (2010)
	Arabidopsis β‐expansin	Arabidopsis: CaMV 35S	Acetylxylan esterase (3.1.1.72) Rhamnogalacturonan acetyl esterase (3.1.1.86) Feruloyl esterase (3.1.1.73)	Pogorelko *et al*. (2011, 2013)
	BAASS	Arabidopsis: CaMV 35S Maize: Rubi3 Maize Seeds: Glb1, Glb2 + Glb1 + pr26, Rice Glutelin, Rubi3 Tobacco: Pact2 Wheat Seeds: Wheat Glutenin 1DX5	Endo‐1,4‐β‐glucanase (3.2.1.4) Cellulose 1,4‐β‐cellobiosidase^R^ (3.2.1.176) Cellulose 1,4‐β‐cellobiosidase^NR^ (3.2.1.91) Endo‐1,4‐β‐xylanase (3.2.1.8) Feruloyl esterase (3.1.1.73) Manganese peroxidase (1.11.1.13)	Borkhardt *et al*. (2010), Clough *et al*. (2006), Devaiah *et al*. (2013), Egelkrout *et al*. (2013), Gray *et al*. (2011b), Hahn *et al*. (2014), Harholt *et al*. (2010), Hood *et al*. (2007), Shen *et al*. (2012)
	CALSP	Tobacco: CaMV 35SS	Endo‐1,4‐β‐glucanase (3.2.1.4) Cellulose 1,4‐β‐cellobiosidase^NR^ (3.2.1.91)	Jiang *et al*. (2011)
	Chitinase 1	Tobacco: CaMV 35S	Endo‐1,4‐β‐mannosidase (3.2.1.78)	Hoshikawa *et al*. (2012)
	Maize expansin B	Brachypodium: Maize Ubiquitin	Acetyl xylan esterase (3.1.1.72 Rhamnogalacturonan acetyl esterase (3.1.1.86)	Pogorelko *et al*. (2013)
	MMA	Tobacco: CaMV 35SS, alcAmin35S	Endo‐1,4‐β‐glucanase (3.2.1.4)	Klose *et al*. (2013, 2015, 2012)
	PPI	Potato: CaMV 35SS Tall Fescue: Rice Actin 1, LmSee1	Endo‐1,4‐β‐xylanase (3.2.1.8) Feruloyl esterase (3.1.1.73)	Buanafina *et al*. (2015, 2012, 2010), Yang *et al*. (2007)
	PR‐S; Pr1a; Pr1b	Alfalfa: tCUP4 Maize: CaMV 35S Maize Seeds: Glb1 Rice: CaMV 35S, Mac Tobacco: Mac, RbcSK‐1A, CaMV 35S, CaMV 35SS	Endo‐1,4‐β‐glucanase (3.2.1.4) Cellulose 1,4‐β‐cellobiosidase^R^ (3.2.1.176) Polygalacturonase (3.2.1.15) Feruloyl esterase (3.1.1.73)	Badhan *et al*. (2014), Biswas *et al*. (2006), Chou *et al*. (2011), Dai *et al*. (2005), Mahadevan *et al*. (2011), Oraby *et al*. (2007), Park *et al*. (2011b), Pereira *et al*. (2014), Ransom *et al*. (2007)
	PttCel9B3	Hybrid Aspen: CaMV 35S	4‐O‐methyl‐glucuronoyl methylesterase (3.1.1.‐)	Latha Gandla *et al*. (2015)
	PvPGIP1	Arabidopsis: OlexA‐46, SAG12	Polygalacturonase (3.2.1.15) Pectate Lyase (4.2.2.2)	Tomassetti *et al*. (2015)
	Rice α‐amylase; Rice α‐amylase 3A; Rice α‐amylase 3D	Sunflower: CaMV 35S Tobacco: CaMV 35S; Pact2	Endo‐1,4‐β‐glucanase (3.2.1.4) Endo‐1,4‐β‐xylanase (3.2.1.8)	Hahn *et al*. (2014), Hwang *et al*. (2012) and Jung *et al*. (2014)
Endoplasmic reticulum	ALE/LKDEL	Tall Fescue: Rice Actin 1, Soya bean Heat Shock	Feruloyl esterase (3.1.1.73)	Buanafina *et al*. (2010)
	Arabidopsis 2S2/DIKDEL	Tobacco: MMV	Endo‐1,4‐β‐xylanase (3.2.1.8) Feruloyl esterase (3.1.1.73)^a^	Chatterjee *et al*. (2010)
	BAASS/KDEL	Maize Seeds: Glb1 Tobacco: CaMV 35S Wheat Seeds: Wheat Glutenin 1DX5	Endo‐1,4‐β‐glucanase (3.2.1.4) Cellulose 1,4‐β‐cellobiosidase^R^ (3.2.1.176) Endo‐1,4‐β‐xylanase (3.2.1.8) Feruloyl esterase (3.1.1.73)	Harholt *et al*. (2010), Hood *et al*. (2007) and Llop‐Tous *et al*. (2011)
	CALSP/HDEL	Tobacco: CaMV 35SS	Endo‐1,4‐β‐glucanase (3.2.1.4) Cellulose 1,4‐β‐cellobiosidase^NR^ (3.2.1.91)	Jiang *et al*. (2011)
	MMA/KDEL	Tobacco: CaMV 35SS	Endo‐1,4‐β‐glucanase (3.2.1.4)	Klose *et al*. (2015, 2012)
	Pr1b/KDEL	Alfalfa: tCUP4 Tobacco: CaMV 35SS	Polygalacturonase (3.2.1.15) Feruloyl esterase (3.1.1.73)	Badhan *et al*. (2014) and Pereira *et al*. (2014)
	SPER/KDEL	Maize: RbcS1 Tobacco: Mac	Endo‐1,4‐β‐glucanase (3.2.1.4)	Dai *et al*. (2005), Mei *et al*. (2009) and Park *et al*. (2011b)
	γ‐zein/SEKDEL	Sugarcane: Maize PepC, Maize Ubiquitin 1	Endo‐1,4‐β‐glucanase (3.2.1.4) Cellulose 1,4‐β‐cellobiosidase^R^ (3.2.1.176) Cellulose 1,4‐β‐cellobiosidase^NR^ (3.2.1.91)	Harrison *et al*. (2011, 2014b)
Chloroplast	–	Tobacco: Prrn, PpsbA, PpsbA + T7g10	Endo‐1,4‐β‐glucanase (3.2.1.4) Cellulose 1,4‐β‐cellobiosidase^NR^ (3.2.1.91) 1,4‐β‐glucosidase (3.2.1.21) Xyloglucan‐specific Endo‐1,4‐β‐glucanase (3.2.1.151) Endo‐1,4‐β‐xylanase (3.2.1.8) Endo‐1,4‐β‐mannosidase (3.2.1.78) Pectate lyase (4.2.2.2) Pectin lyase (4.2.2.10) Acetyl xylan esterase (3.1.1.72) Lipase (3.1.1.3) Cutinase (3.1.1.74) Swollenin Manganese peroxidase (1.11.1.13)	Agrawal *et al*. (2011), Espinoza‐Sánchez *et al*. (2015), Gray *et al*. (2009), Gray *et al*. (2011c), Jin *et al*. (2011), Kim *et al*. (2011), Kolotilin *et al*. (2013), Nakahira *et al*. (2013), Pantaleoni *et al*. (2014), Petersen and Bock (2011), Verma *et al*. (2013, 2010), Yu *et al*. (2007) Ziegelhoffer *et al*. (2009)
	CAB	Tobacco: RbcSK‐1A	Endo‐1,4‐β‐glucanase (3.2.1.4)	Kim *et al*. (2010)
	CTP	Tobacco: Pact2	Cellulose 1,4‐β‐cellobiosidase^NR^ (3.2.1.91)	Hahn *et al*. (2014)
	*Cyanophora paradoxa* FNR	Sugarcane: Maize PepC	Endo‐1,4‐β‐glucanase (3.2.1.4)	Harrison *et al*. (2011, 2014b)
	RbcS; RbcS‐2A	Arabidopsis: CaMV 35S Tobacco: Mac, RbcS‐3C, RbcSK‐1A, MRbcSK‐1A, MRbcSK‐1A + aps, CaMV 35S	Endo‐1,4‐β‐glucanase (3.2.1.4) Cellulose 1,4‐β‐cellobiosidase^NR^ (3.2.1.91) 1,4‐β‐glucosidase (3.2.1.21) Endo‐1,4‐β‐xylanase (3.2.1.8) Polygalacturonase (3.2.1.15)	Dai *et al*. (2005), Jung *et al*. (2013), Kim *et al*. (2010), Lee *et al*. (2012), Pereira *et al*. (2014)
	Rubisco Activase	Alfalfa: tCUP4 Arabidopsis: RbcSK‐1A, CaMV 35S Tobacco: RbcSK‐1A, CaMV 35S	Endo‐1,4‐β‐glucanase (3.2.1.4) 1,4‐β‐glucosidase (3.2.1.21) Endo‐1,4‐β‐xylanase (3.2.1.8) Feruloyl esterase (3.1.1.73)	Badhan *et al*. (2014), Bae *et al*. (2006, 2008), Jung *et al*. (2010), Kim *et al*. (2010), Lee *et al*. (2012) and Mahadevan *et al*. (2011)
Vacuole	ALE; ALE/Frameshift KDEL	Italian Ryegrass: Rice actin Tall Fescue: Rice actin 1, Maize Ubiquitin, Soya bean Heat Shock, LmSee1	Endo‐1,4‐β‐xylanase (3.2.1.8) Feruloyl esterase (3.1.1.73)	Buanafina *et al*. (2015, 2012, 2008, 2006)
	Maize Proaleurain + VT	Maize Seeds: Zm‐leg1A	Endo‐1,3(4)‐β‐glucanase (3.2.1.6) Endo‐1,4‐β‐mannosidase (3.2.1.78) α‐Galactosidase (3.2.1.22)	Xu *et al*. (2013), Yang *et al*. (2015) and Zhang *et al*. (2013)
	Pr1b/CTPP	Alfalfa: tCUP4 Tobacco: CaMV 35SS	Polygalacturonase (3.2.1.15) Feruloyl esterase (3.1.1.73)	Badhan *et al*. (2014) and Pereira *et al*. (2014)
	Sweet Potato Sporamin A	Tobacco: Mac	Endo‐1,4‐β‐glucanase (3.2.1.4)	Dai *et al*. (2005)
	VT	Maize: CaMV 35S Maize Seeds: Glb1	Endo‐1,4‐β‐glucanase (3.2.1.4) Cellulose 1,4‐β‐cellobiosidase^R^ (3.2.1.176) 1,4‐β‐glucosidase (3.2.1.21)	Hood *et al*. (2012, 2007, 2014) and Park *et al*. (2011b)
	γ‐zein/DELKAEAK	Maize: Maize PepC Sugarcane: Maize PepC;	Endo‐1,4‐β‐glucanase (3.2.1.4) Cellulose 1,4‐β‐cellobiosidase^R^ (3.2.1.176) Cellulose 1,4‐β‐cellobiosidase^NR^ (3.2.1.91)	Harrison *et al*. (2014a, 2011, 2014b)
Mitochondria	Cox IV	Maize: RbcS1	Endo‐1,4‐β‐glucanase (3.2.1.4)	Mei *et al*. (2009)
Golgi System	RST; RST/Frameshift KDEL	Tall Fescue: Rice Actin 1	Endo‐1,4‐β‐xylanase (3.2.1.8) Feruloyl esterase (3.1.1.73)	Buanafina *et al*. (2012, 2010)
Peroxisome	‐/SKL	Arabidopsis: RbcSK‐1A	Endo‐1,4‐β‐xylanase (3.2.1.8)	Bae *et al*. (2006)
Endosperm	Glub‐4	Maize Seeds: Glub‐4	Endo‐1,4‐β‐xylanase (3.2.1.8)	Gray *et al*. (2011b)
Chloroplast & Peroxisome	RA/SKL	Arabidopsis: RbcSK‐1A	Endo‐1,4‐β‐xylanase (3.2.1.8)	Bae *et al*. (2006)

^R^Reducing end cellobiohydrolase.

^NR^Non‐reducing end cellobiohydrolase.

*alc*Amin35S, alcohol‐inducible promoter based on CaMV 35S; ALE, barley aleurain vacuole‐targeting signal; aps, amplification promoting sequence; BAASS, Barley α‐amylase signal sequence; CAB, Chlorophyll *a‐/b*‐binding protein; CALSP, tobacco calreticulin signal peptide; CaMV35SS, double CaMV 35S promoter; Cox IV, Yeast cytochrome c oxidase subunit; CTP, artificial dicot chloroplast targeting sequence; CTPP, C‐terminal propeptide tobacco chitinase vacuolar sorting signal; DELKAEAK, vacuole sorting determinant; FNR, ferredoxin‐NADP^+^‐oxidoreductase; Frameshift KDEL, frameshifted terminal peptide (ETTEG) that removes ER retention; Glb1, Maize globulin1; Glb2, globulin2; Glub‐4, rice glutelin B‐4 gene; Gt1, rice glutelin Gt1 promoter; (SE/DI/L)KDEL/HDEL, endoplasmic reticulum retention signal; LmSee1, Lolium multiflorum senescence‐enhanced gene promoter; Mac, hybrid of Ti plasmid mannopine synthetase promoter and cauliflower mosaic virus 35S promoter enhancer; MMA, leader peptide derived from murine monoclonal antibody mAb24; MMV, Mirabilis mosaic virus promoter; MRbcSK‐1A, three alfalfa RbcS promoters (RbcSK‐1A) without negative regulatory region; OlexA‐46, β‐estradiol‐inducible promoter; Pact2, Arabidopsis actin 2 promoter; PepC, phosphoenolpyruvate carboxylase; PpsbA, PSII protein D1 promoter; PPI, Potato protease inhibitor II; Prrn, Tobacco 16S ribosomal ERNA promoter; PR‐S/PR1a/PR1b, pathogenesis‐related proteins; PvPGIP1, *P. vulgaris* polygalacturonase‐inhibiting protein; RA, Rubisco activase; RbcS, Rubisco small subunit; Rice SGR, Rice Stay Green gene; RST, rat sialyl transferase Golgi targeting motif; Rubi3, rice ubiquitin promoter; SAG12, Arabidopsis senescence‐inducible promoter; SKL, peroxisome‐targeting C‐terminal sequence; SPER, endoplasmic reticulum targeting signal peptide; T7g10, bacteriophage T7 gene 10 N‐terminal enhancer; VT, vacuole‐targeting signal peptide; Zm‐leg1A, maize legumin promoter.

a Multifunctional enzyme.

### Targeting the apoplast for enzyme accumulation

The apoplast is a free diffusional space outside of the plasma membrane that includes the cell wall. A number of different signal peptides have been used to target cell wall‐deconstructing enzymes to the apoplast (Table 1); however, the two most widely used are barley α‐amylase (BAASS) and the pathogenesis‐related proteins (PR‐S/Pr1a/Pr1b). BAASS is often used in conjunction with a seed‐specific promoter for protein accumulation in cereal seeds, although it has also been used with Arabidopsis and tobacco (Borkhardt *et al*., 2010; Hahn *et al*., 2014). Apoplast targeting using pathogenesis‐related protein signal peptides has been successfully accomplished in tobacco (Dai *et al*., 2005; Mahadevan *et al*., 2011; Park *et al*., 2011b; Pereira *et al*., 2014), maize (Biswas *et al*., 2006; Park *et al*., 2011b; Ransom *et al*., 2007), rice (Chou *et al*., 2011; Oraby *et al*., 2007) and alfalfa (Badhan *et al*., 2014), without disturbing cell wall integrity and with reasonable enzyme yields (1.2%–6.1%) (Table S1).

When expressed at low levels, heterologous enzymes targeted to the apoplast show no apparent negative impacts on plant growth and development (Ziegelhoffer *et al*., 2001). However, at high levels of expression, biologically active cell wall‐deconstructing enzymes that are targeted to the apoplast can directly affect the plant cell wall, resulting in changes to cell wall composition (Pogorelko *et al*., 2011). In some cases, these changes are beneficial, reducing cell wall recalcitrance (Badhan *et al*., 2014; Brunecky *et al*., 2011; Buanafina *et al*., 2010; Pogorelko *et al*., 2011, 2013), and in others, the enzymes negatively impact plant growth and development (Buanafina *et al*., 2015; Chou *et al*., 2011; Klose *et al*., 2013, 2015).

### Targeting the ER for enzyme accumulation

The ER is a suitable location for the accumulation of heterologous enzymes because it contains a series of molecular chaperones, such as the ER luminal binding protein, that is needed during protein folding and assembly, and which prevents the transport of immature protein molecules (Valente *et al*., 2009). Targeting of heterologous enzymes into the ER is accomplished by adding a retention signal such as KDEL (Harholt *et al*., 2010; Hood *et al*., 2007; Klose *et al*., 2012, 2015; Park *et al*., 2011b; Pereira *et al*., 2014), HDEL (Jiang *et al*., 2011), SEKDEL (Harrison *et al*., 2011), DIKDEL (Chatterjee *et al*., 2010) or LKDEL (Buanafina *et al*., 2010) to the C‐terminal of the protein sequence. Nullification of ER retention can be accomplished by attaching a Frameshift KDEL (ETTEG) to the C‐terminus, which allows the enzymes to be expressed in other compartments (Buanafina *et al*., 2006, 2008, 2010). When E1 (AcCel5A) was targeted into the ER of maize stover, the yield was much higher than E1 targeted to the mitochondria (Mei *et al*., 2009). Another report confirms the suitability of ER targeting for high‐level production of heterologous enzymes (E1/AcCel5A, CBHI/TrCel7A) in maize seeds (Hood *et al*., 2007).

### Targeting the vacuole for enzyme accumulation

Vacuoles are storage reservoirs for water and starch within the cell and account for ~30%–90% of plant cell volume depending on plant maturity (Alberts *et al*., 2007). Although the number of vacuoles per plant cell is relatively low, the large volume potentially occupied by vacuoles within the cell is beneficial for high enzyme accumulation. Barley aleurain vacuole‐targeting signal (ALE), sweet potato sporamin A and vacuole‐targeting signal peptide (VT) have all been used as signal peptides to accumulate endoglucanases, cellobiohydrolases, β‐glucosidases and endoxylanases in plant vacuoles (Table 1). Vacuole targeting can also be facilitated by including, at the 3′ end of the construct, a vacuole sorting determinant such as DELKAEAK (Harrison *et al*., 2011, 2014a,b) or a carboxyl‐terminal propeptide (Badhan *et al*., 2014; Pereira *et al*., 2014). In general, the accumulation and retention of enzyme activity tends to be high within vacuoles. For both green and senescent tissues, synthetic CBHI/Cel7 and CBHII/Cel6 showed higher activities when targeted to sugarcane leaf vacuoles compared to targeting to the ER (Harrison *et al*., 2011). And in maize seeds, the vacuole accumulated the highest amount of E1/AcCel5A of all the organelles (Hood *et al*., 2007). The exception is one case where the accumulation of enzymes within the vacuole was significantly lower compared to the ER, chloroplast and apoplast (Dai *et al*., 2005).

### Targeting chloroplasts for enzyme accumulation via nuclear transformation

The chloroplast is considered particularly suitable for production and sequestration of enzymes because of their large copy number within the cell and because their double‐membrane provides added protection against interference by the sequestered enzymes with cellular growth and metabolic activity. For chloroplast targeting, the two most common signal peptides are Rubisco activase (RA) and Rubisco small subunit (RbcS). Production of endoglucanase TmCel5A in tobacco chloroplasts using the RbcS signal peptide gave much higher yields compared with other signal peptides including light‐harvesting chlorophyll a‐/b‐binding protein (CAB) and RA (Kim *et al*., 2010). *Cyanophora paradoxa* ferredoxin‐NADP^+^‐oxidoreductase (FNR) has also been used for chloroplast targeting in sugarcane, though with very low yields (<0.05% TSP) (Harrison *et al*., 2011, 2014b).

### Targeting chloroplasts for enzyme accumulation via plastid transformation

Because chloroplasts contain their own genetic material, instead of using signal peptides it is also possible to insert the gene of interest directly into the plastid genome via homologous recombination. Plastid transformation has a number of distinct advantages over nuclear transformation. First, because individual cells contain many more copies of the plastid genome compared with the nuclear genome, it is possible to produce high copy numbers of the transgene and increase protein accumulation in transgenic plants (Hanson *et al*., 2013). Other advantages of plastid transformation include site‐specific targeting of the transgene without disrupting essential coding and noncoding regions, the ability to express multiple proteins from polycistronic mRNA at high levels, the absence of gene silencing and more secure containment of the transgene due to maternal transfer of the plastid genome (Chen *et al*., 2014; Egelkrout *et al*., 2012; Heifetz, 2000; Kim *et al*., 2011; Wani *et al*., 2010).

Accumulation of enzymes can be fairly high within the chloroplast, with a number of studies achieving over 10% total soluble protein accumulation (Gray *et al*., 2009; Gray *et al*., 2011c; Hahn *et al*., 2014; Kim *et al*., 2011; Ziegelhoffer *et al*., 2009), with the highest levels from plastid genome transformation. Within the same study, enzyme accumulation in chloroplasts is generally higher compared with accumulation in other organelles, including the apoplast, cytosol, vacuoles, ER and peroxisomes (Bae *et al*., 2006, 2008; Harrison *et al*., 2011; Mahadevan *et al*., 2011). However, high expression of enzymes within chloroplasts can occasionally cause pigment and photosynthetic deficiencies (Agrawal *et al*., 2011; Kolotilin *et al*., 2013; Nakahira *et al*., 2013; Pogorelko *et al*., 2011; Verma *et al*., 2013), although these deficiencies does not always result in negative impacts on growth or reproduction (Agrawal *et al*., 2011). These phenotypic and physiological defects can be partially overcome by generating heteroplasmic plants that contain a mix of transgenic and nontransgenic plasmids (Pogorelko *et al*., 2011).

### Targeting mitochondria for enzyme accumulation

Mitochondria have the potential to be highly suitable compartments for enzyme production. This is because, like chloroplasts, mitochondria are present at high copy numbers within the cell and are surrounded by a double‐layered membrane that increases the likelihood of enzyme containment, preventing premature cell wall degradation. However, although mitochondria also contain their own genetic material, plastid transformation is a demonstrated technology while mitochondrial genome transformation has never been successfully achieved in land plants (Colas des Francs‐Small and Small, 2014). Nuclear transformation coupled with the use of signal peptides is the only method currently available to target the mitochondria. A signal peptide derived from yeast cytochrome c oxidase subunit (Cox IV) has been used to target E1/AcCel5A into maize mitochondria (von Heijne, 1986; Mei *et al*., 2009). However, the amount of enzyme accumulated within the mitochondria was on average lower than that obtained by targeting into the ER, and more fermentable sugars were produced when the ER‐targeted transgenic plant extract was used to deconstruct lignocellulosic biomass (Mei *et al*., 2009). Only a single study has been conducted on the use of mitochondria for enzyme accumulation, and enzyme yields were quite low (0.2% yield TSP of endoglucanase), so it is unknown whether higher enzyme accumulation in mitochondria would significantly disrupt respiration, similar to how high enzyme accumulation in chloroplasts can disrupt photosynthesis.

### Targeting peroxisomes for enzyme accumulation

Peroxisomes are small, ubiquitous organelles that are involved in fatty acid degradation, carbon metabolism and pathogen defence within plant cells (Hu *et al*., 2012). A number of signal peptides are available that target peroxisomes (Lingner *et al*., 2011); however, it is also possible to accumulate enzymes in peroxisomes by attaching a peroxisome‐targeting termination sequence, SKL, to the C‐terminal. Using this sequence, endoxylanase (XylII/TrXyn11A) from *Trichoderma reesei* was successfully targeted to and accumulated in peroxisomes of transgenic Arabidopsis (Bae *et al*., 2006).

## Strategies to increase the expression of cell wall‐deconstructing enzymes in plants

### Promoters for enhanced accumulation of enzymes

A number of techniques have been employed in an attempt to boost the expression of cell wall‐deconstructing enzymes *in planta*, and choice of promoter is particularly important.

A variety of promoters are available (Saunders *et al*., 2001) and have been used for heterologous enzyme production, including constitutive, tissue‐ and organelle‐specific, time‐regulated‐based on the developmental phase, or chemically inducible (Figure 1b). Promoters can be subdivided into two classes, those derived from monocot species and those derived from dicot species (or from a viral vector that targets monocot or dicot species). A promoter is generally more effective when expressed in the same class of plant from which it was derived (Jang *et al*., 2002; Park *et al*., 2010; Schäffner and Sheen, 1991). One of the most common constitutive promoters is the cauliflower mosaic virus (CaMV) 35S promoter. The 35S promoter has been used to produce a variety of enzymes in dicots: duckweed (0.24% TSP), tobacco (0%–9% TSP), potato (5% TSP), Arabidopsis (1.4%–14% TSP), hybrid aspen (N/A) and sunflower (0.05%–0.07% TSP); and monocots: rice (4.9% TSP) and maize (0.9%–3.1% TSP) (Tables 1 and S1). However, it is generally more effective in dicots (Park *et al*., 2010). Several efforts have been made to improve the activity of 35S in monocot species. One strategy involved modifying the 35S promoter by adding an intron between the promoter and the open reading frame of transgene, which improved promoter activity in monocots, including some grasses (Fischer and Schillberg, 2006). The activity of the 35S promoter can be further enhanced by duplication or when combined with an enhancer region. One example is the Mac promoter, a hybrid of a mannopine synthetase promoter and the CaMV 35S promoter enhancer. Use of the Mac promoter increased the heterologous E1/AcCel5A yield in tobacco by 20‐fold compared with other promoters (Chou *et al*., 2011). Although the CaMV 35S promoter has been used for enzyme expression in monocots, there are a number of constitutive monocot‐specific promoters that have also been used including maize ubiquitin, rice ubiquitin (rubi3) and rice actin (Figure 1b). For a thorough list of potential promoters for protein expression in plants, we refer the reader to the review by Egelkrout *et al*. (2012).

### Promoters for tissue‐ and organ‐specific accumulation of enzymes

A few tissue‐specific promoters have been investigated for the production of heterologous enzymes in plants, including leaf‐specific light‐inducible promoters based on of Rubisco small subunit genes (dicot: RbcSK‐1A, RbcS‐3C; monocot: RbcS1) (Table 1 and Figure 1b). The rbcS1 promoter derived from chrysanthemum has been shown to be advantageous compared with other promoters because of its higher gene expression levels, as much as 7–8 fold higher than the commonly used constitutive 35S promoter (Outchkourov *et al*., 2003). Other monocot tissue‐specific promoters that have been used include the maize mesophyll‐specific promoter (phosphoenolpyruvate carboxylase or PepC), and a variety of endosperm and embryo‐specific promoters: maize globulin (Glb1 & Glb2), maize legumin (Zm‐leg1A), rice glutelin (rice glutelin & Glub‐4) and wheat glutenin (1DX5). Seed‐specific promoters in particular are highly effective and result in some of the highest levels of protein accumulation (3.2%–30% TSP) (Table S1). Enzymes can also be stored in seeds postharvest and still retain activity after long‐term storage at room temperature (Zhang *et al*., 2012). High enzyme yields are also achieved using a chloroplast‐specific promoter, either tobacco 16S ribosomal ERNA promoter (Prrn) or PSII protein D1 promoter (PpsbA) in combination with flanking sequences (Agrawal *et al*., 2011; Gray *et al*., 2009; Jin *et al*., 2011; Kim *et al*., 2011; Kolotilin *et al*., 2013; Verma *et al*., 2010, 2013; Yu *et al*., 2007; Ziegelhoffer *et al*., 2009). These promoters support targeted integration of the gene of interest into the chloroplast genome via homologous recombination, as described in the previous section.

### Promoters for inducible expression of enzymes

Chemical, temperature and senescence‐inducible promoters are also of interest as they allow the production of enzymes just prior to biomass harvest, and avoid early production of enzymes that can cause negative phenotypic impacts. One study used an alcohol‐inducible promoter that is based on of the CaMV 35S promoter (*alc*Amin35S) and showed increased enzyme expression levels without any adverse effects on the plant (Klose *et al*., 2013). A β‐estradiol‐inducible promoter (OlexA‐46) was used to trigger expression of pectate lyase in Arabidopsis; however, high levels of the transgene caused reductions in growth even when no inducer was applied (Tomassetti *et al*., 2015). Senescence‐inducible promoters that trigger enzyme accumulation immediately upon cessation of growth have been used to provide a great effect in a number of studies. The rice stay green promoter has been successfully used to induce expression of a cellobiohydrolase in rice straw at the onset of senescence and also successfully eliminated the negative phenotype observed in constitutively expressed plants (Furukawa *et al*., 2014). The senescence‐inducible promoter SAG12 expressed polygalacturonase (pga2) only during the late stages of Arabidopsis development with no negative impacts to plant growth (Tomassetti *et al*., 2015). LmSee1, similar to maize P_SEE1_ (Robson *et al*., 2004), has been used to increase ferulic acid esterase and xylanase expression in tall fescue (Buanafina *et al*., 2008, 2010, 2012, 2015). The use of this senescence‐inducible promoter resulted in higher heterologous enzyme activity compared with the constitutive promoters: ubiquitin, CaMV 35S and rice actin (Buanafina *et al*., 2008).

### Use of multi‐organelle targeting

Besides promoter selection, other strategies have been employed to increase the expression of heterologous enzymes in plants. Multi‐organelle targeting can be accomplished by incorporating a signal peptide for one organelle with a C‐terminal targeting sequence for a second organelle. Using this method, xylanase was targeted to both chloroplasts and peroxisomes, and enzyme accumulation was significantly higher than when it was targeted to each organelle individually (Bae *et al*., 2006). Another means to achieve multi‐organelle targeting is by taking advantage of alternative splicing, a regulated process that naturally occurs in eukaryotes where the exons from a single gene naturally recombine in different ways, producing different proteins. In this case, 5′ mRNA tags are used that can recombine to different acceptor sites during alternative splicing events. When employed, this strategy successfully enabled a reporter protein to be targeted to chloroplasts, peroxisomes and the cytosol (Voges *et al*., 2013). Another possibility is to embed the second targeting sequence within the first, which reduces the possibility of expression in nontargeted compartments (Voges *et al*., 2013).

### Attaching promoter amplification sequences and multiple copies of transcription units

Increasing the copy number of the promoter + transcription unit within a single gene cassette can also increase the yield of plant‐produced enzymes. For all constructs tested, those with multiple copies of the enzyme coding region within the construct had consistently higher activity of Cel5A (E1) or CBHI than those with a single copy (Egelkrout *et al*., 2013).

In some cases, increased enzyme expression can also be obtained by attaching a cis‐acting element amplification sequence upstream of the promoter. However, the success of this method in increasing enzyme yields is dependent on the amplification sequence being used, the enzyme being expressed, and the organelle that is being targeted for expression. Fusion of an amplification sequence upstream of a modified RbcsK‐1A promoter increased β‐glucosidase (TmBgl3) production in tobacco chloroplasts by 2% compared with the nonamplified promoter (Jung *et al*., 2013). However, in another study on xylanase production in tobacco chloroplasts, the effect of the amplification sequence depended on the enzyme being expressed. Incorporation of the N‐terminal enhancer T7g10 increased production of AnXyn11A (from 2.5% to 6.0% TSP), but decreased production of AnXyn10A (from 3.3% to 0.2% TSP) (Kolotilin *et al*., 2013). Another study observed an improvement only with certain amplification sequences in certain tobacco organelles. Fusion of certain proteins including elastin‐like polypeptide (ELP) repeats or hydrophobin (HFBI) has the ability to both increase heterologous protein expression and facilitate their purification (Conley *et al*., 2009; Joensuu *et al*., 2010). Incorporation of an ELP repeat into the construct increased polygalacturonase expression in vacuoles, but not in the ER or apoplast, while fusion with HFBI impaired both accumulation and activity of the enzyme (Pereira *et al*., 2014). Two possibilities were given for the poor performance of HFBI in this study, in that its shape interfered with proper enzyme folding and led to ER‐associated protein degradation, or that the linker between HFBI and the enzyme was not optimized, which could impact activity and yield (Pereira *et al*., 2014).

### Chloroplast‐specific strategies

A number of strategies have been specifically developed to increase the heterologous enzyme yields in chloroplasts. One strategy, as mentioned previously, is to use a plastid‐specific promoter to take advantage of the high plastid genome copy number. It is also possible to increase chloroplast enzyme expression by altering the sequence of the downstream box (DB) region. In one study, the level of endoglucanase expression in tobacco plastids showed a 100‐fold difference in yield between three different DB regions (Gray *et al*., 2009), although the results are enzyme dependent and show a different pattern for β‐glucosidase expression (Gray *et al*., 2011c).

### Use and manipulation of high‐yield germplasm

Selection of an appropriate host species for genetic transformation is based on a number of factors including ease of transformation, availability and effectiveness of suitable promoters, stability of transformation within the species, and biomass yield of the crop plant. However, even within the same species biomass yields can vary widely based on field and environmental conditions and genetic background. Once a host species is selected, using appropriate genetic backgrounds for transformation is very important as it can significantly impact the accumulation of heterologous cell wall‐deconstructing enzymes. By inserting the desired cell wall‐deconstructing genes into high oil maize germplasm and targeting seeds for expression, the amount of enzymes produced increased dramatically, with AcCel5A (E1) increasing by 50%, manganese peroxidase by 2‐fold, and CBHI by 5‐fold compared with standard or elite genotypes (Clough *et al*., 2006; Hood *et al*., 2012). Using a high biomass cultivar for production of xylanases in tobacco also increased production by 60% compared with a standard cultivar (Kolotilin *et al*., 2013). Hybridizing transgenic plants is another way to increase yields, and in an initial field study, there were no differences between a transgenic hybrid and a conventional wild‐type hybrid (Garda *et al*., 2015).

## Expanding the types and numbers of enzymes targeted for accumulation *in planta*

A large set of enzyme activities is needed in order to effectively deconstruct the variety of polymeric sugars within the plant cell wall (Supporting Information; Figures S1–S4). These enzymes function synergistically in a mixture and the addition of even small amounts of minor enzyme components is known to significantly improve enzymatic hydrolysis yields (Banerjee *et al*., 2010; Gao *et al*., 2011; Jabbour *et al*., 2014). Prior to 2005, the expression of cell wall‐degrading enzymes in plants was limited to a relatively small number of thermostable enzymes, with an even more limited range of enzyme activities: two endoglucanases and a cellobiohydrolase (Dai *et al*., 2000; Jin *et al*., 2003; Teymouri *et al*., 2004; Ziegelhoffer *et al*., 1999, 2001; Ziegler *et al*., 2000), a handful of endoxylanases (Herbers *et al*., 1995; Kimura *et al*., 2003; Leelavathi *et al*., 2003) and a couple of accessory glycoside hydrolases (Montalvo‐Rodriguez *et al*., 2000). Over the past 10 years, although the most commonly studied enzyme has remained a thermostable endoglucanase (E1/AcCel5A) from *Acidothermus cellulolyticus*, the number of enzymes that have been evaluated has expanded significantly, both in terms of activities covered and organism of origin. The list now includes, not just endoglucanases and endoxylanases, but also a large variety of backbone and side‐chain cleaving glycoside hydrolases, carbohydrate esterases, and even laccases and peroxidases, which target lignin and other polyphenolics (Tables 1 and S2).

### Expression of multiple enzyme activities within a single plant

One strategy to accommodate the required enzyme synergy is to generate individual enzymes in separate lines of transgenic plants and then recombine them or their extracts during hydrolysis (Verma *et al*., 2010). Ultimately however, it may be desirable and more efficient to produce multiple enzyme activities within a single plant. There are a number of strategies that have been employed to accomplish this. One method is to perform sequential transformations on a single transformant and in this way incorporate the genes for more than one enzyme within the genome (Buanafina *et al*., 2015). A stack of genes for multiple enzymes can also be delivered into the plant genome at one time via Agrobacterium‐mediated transformation and biolistic bombardment (Que *et al*., 2010). Another successful approach linked two enzymes, each with a different signal peptide, via the 2A self‐cleaving oligopeptide from foot‐and‐mouth disease virus (Lee *et al*., 2012). 2A self‐cleaves *in vivo* and then the separate enzymes are directed by their appropriate signal peptides to their target compartments (Lee *et al*., 2012). This means that this method could potentially be used to target different enzymes to different subcellular compartments.

Another option, instead of trait‐stacking the genes for multiple enzymes, is to express multifunctional enzymes. While many cell wall‐deconstructing enzymes are active on only one substrate, multifunctional enzymes, sometimes called ‘chimaeras’, possess more than one enzymatic activity and can be either naturally occurring or synthetically produced. These enzymes have multiple activities either because they possess an active site that is able to accommodate more than one substrate, or they have multiple active sites, each of which has activity towards a different substrate (Cho *et al*., 2006; Elleuche, 2015; Fan and Yuan, 2010; Ferrer *et al*., 2012). If the enzyme that is chosen for expression has activity towards multiple substrates, this reduces the number of genes needed and the complexity of the transformation (Fan and Yuan, 2010).

## Problems and pitfalls in enzyme expression

### Enzyme truncation due to the loss of carbohydrate binding modules

Carbohydrate binding modules (CBMs) occur in many different types of enzymes and are small protein subunits that are attached by a linker to the catalytic domain of an enzyme and facilitate binding and movement of the enzyme along the polysaccharide. Many types of enzymes show enhanced activity when linked to a CBM (Hervé *et al*., 2010; Park *et al*., 2011a), even those that are not linked to one naturally (Reyes‐Ortiz *et al*., 2013; Telke *et al*., 2012). Unfortunately, even though a full gene including the CBM is inserted into a plant genome, in many cases the enzymes that are produced are truncated to only include the catalytic domain or the catalytic domain and the linker (Dai *et al*., 2005; Harrison *et al*., 2011; Hood *et al*., 2007; Klose *et al*., 2013, 2015; Sun *et al*., 2007). In order to avoid removal of the CBM by endogenous proteases, a number of different strategies have been employed. For endoglucanases, it is possible to simply avoid truncation by choosing an enzyme that is mono‐domain and does not have a CBM (Harrison *et al*., 2011). Alternatively, targeting enzymes with CBMs to the ER and chloroplast can have a limited benefit with partial (though in many cases minor) retention of intact proteins (Dai *et al*., 2005; Klose *et al*., 2015; Mahadevan *et al*., 2011). Another strategy that has successfully produced a fully intact enzyme in chloroplasts involved fusing an EG catalytic domain (TmCel5A) from *Thermotoga maritima* with CBM6 from *Clostridium stercorarium* (Mahadevan *et al*., 2011). However, it is not clear which characteristics of this fusion protein rendered it more stable to degradation by proteases.

### Phenotypic defects due to enzyme accumulation and interference with cellular function

Phenotypic abnormalities have been observed during plant growth and development due to the accumulation of glycosyl hydrolases, largely occurring when enzymes are accumulated in the apoplast or cytosol. (Phenotypic defects specific to chloroplasts were discussed in the earlier section.) For instance, in one study cellobiohydrolase was constitutively expressed in rice, which resulted in cell wall defects that caused cracks in the leaf surface, and no viable plants were obtained with endoglucanase expression (Nigorikawa *et al*., 2012). In addition to leaf defects, overexpression of endoglucanase and cellobiohydrolase in the cytosol and apoplast in rice has also resulted in yield loss and germination reduction in seeds (Zhang *et al*., 2012), shorter stature and early flowering (Chou *et al*., 2011) and sterility (Nigorikawa *et al*., 2012).

While no phenotypic defects have been shown to occur when xylanases are targeted into the apoplast of dicotyledonous plants (Borkhardt *et al*., 2010; Chatterjee *et al*., 2010; Kimura *et al*., 2010; Yang *et al*., 2007), targeting xylan‐deconstructing enzymes or ferulic acid esterases (FAE) into grass subcellular compartments often, but not always (Xu *et al*., 2013), results in negative effects on plant phenotypes. This is likely due to the importance of xylan and ferulic ester linkages within the grass cell wall compared with dicots (Scheller and Ulvskov, 2010). Significant biomass reduction was observed in wheat seeds that were genetically engineered to overexpress endoxylanase (Harholt *et al*., 2010). In tall fescue, xylanase production reduced plant growth and caused necrotic lesions on the leaves (Buanafina *et al*., 2012). Another study showed severe phenotypic defects in maize including stunting and sterility when overexpressing xylanase under the rice ubiquitin 3 promoter (rubi3) (Gray *et al*., 2011a). Endosperm tissue‐specific expression of endoxylanases and FAE may cause a shrunken seed phenotype in cereals (Gray *et al*., 2011b; Harholt *et al*., 2010). And while FAE expression in grass vegetative tissues showed no phenotypic impact on plant seeds (Buanafina *et al*., 2006, 2008, 2010), this can cause severe reductions in plant growth (Buanafina *et al*., 2015).

### Strategies to avoid enzyme‐triggered phenotypic defects

A couple of strategies have been successfully employed to avoid deleterious impacts of enzyme accumulation. Expression of hyperthermophilic enzymes that have very high optimal temperatures and virtually no activity at ambient temperatures can prevent negative impacts to the cell wall (Borkhardt *et al*., 2010; Herbers *et al*., 1995; Klose *et al*., 2012). As mentioned earlier, it is possible to trigger enzyme expression immediately prior to harvest by using an ethanol‐ or senescence‐inducible promoter (Furukawa *et al*., 2014; Klose *et al*., 2013; Tomassetti *et al*., 2015). Using a senescence‐inducible promoter instead of the constitutive, the negative defects due to cellobiohydrolase expression were completely eliminated (Furukawa *et al*., 2014). Targeting expression to locations other than the cytosol and apoplast can also help prevent phenotypic defects. Constitutive expression of manganese peroxidase in vegetative tissues can cause severe negative phenotypic impacts including cell death and lesions (Clough *et al*., 2006); however, this was not observed when the enzyme was targeted to the chloroplasts (Espinoza‐Sánchez *et al*., 2015), or produced in seeds (Clough *et al*., 2006).

Two methods have been reported for overcoming the deleterious impacts of xylanase accumulation in grasses. These methods include (i) removal of the signal portion of the xylanase gene prior to its transfer into a host plant (Kimura *et al*., 2010), and (ii) incorporation of inteins into enzymes (Shen *et al*., 2012). Inteins are self‐splicing peptides that can be engineered into cell wall‐deconstructing enzymes and used to disrupt proper enzyme function when produced *in planta*. At ambient conditions, the enzymes are inactive, however upon exposure to certain stimuli the protein self‐splices, restoring enzyme function (Shen *et al*., 2012).

## The production and use of heterologous enzymes for biofuel production

### Increasing digestibility by altering cell wall composition

One of the biggest questions related to the production of heterologous cell wall‐deconstructing enzymes *in planta* is how to effectively access them for biofuel production. Some studies have chosen to indirectly use heterologously produced enzymes to increase conversions by altering the biomass composition and cell wall characteristics (Badhan *et al*., 2014; Buanafina *et al*., 2006, 2008, 2010, 2015; Furukawa *et al*., 2013; Latha Gandla *et al*., 2015; Pogorelko *et al*., 2011, 2013; Tsai *et al*., 2012).

### Autohydrolysis of lignocellulosic biomass

Another option is to autohydrolyse the plant biomass using internally generated enzymes (Buanafina *et al*., 2015; Furukawa *et al*., 2014; Nigorikawa *et al*., 2012; Tomassetti *et al*., 2015). In this case, the biomass is ground with no further chemical treatment, and hydrolysed either only using the heterologous enzyme produced within the plant, or in combination with supplemental enzymes. In one study, this was attempted using Cel5A, although the yield was very low (<1% glucose yields) (Mahadevan *et al*., 2011). However, endoglucanase by itself releases little glucose, and cellobiohydrolase and β‐glucosidase are needed to deconstruct cellulose completely. Instead of using monomeric sugar yields that are a poor indicator of endo‐enzyme activity, it is possible to measure the change in molecular weight of the polysaccharides. Autohydrolysis using heterologous thermophilic xylanases (*Dictyoglomus thermophilum* XynA/DtXyn10 and XynB/DtXyn11) lowered the average xylan molecular weight compared with wild‐type plants, indicating increased activity compared with the wild type (Borkhardt *et al*., 2010).

### Extraction of enzymes to avoid pretreatment degradation

Cell wall‐deconstructing enzymes by themselves are generally insufficient to obtain high yields of fermentable sugars, and a thermochemical pretreatment step is necessary to disrupt the cell wall structure and increase enzyme access to the polysaccharides. Many studies have attempted to macerate the biomass and extract the enzymes prior to the pretreatment, in order to prevent their denaturation, and then add them back during enzymatic hydrolysis (Agrawal *et al*., 2011; Harrison *et al*., 2014a; Hood *et al*., 2014; Jung *et al*., 2010; Oraby *et al*., 2007; Pogorelko *et al*., 2011; Ransom *et al*., 2007; Verma *et al*., 2010). However, the extraction of heterologous enzymes adds additional processing and capital costs, particularly if any concentration or filtration is required (Bals and Dale, 2011).

### Direct pretreatment of enzyme‐containing lignocellulosic biomass

An alternative option is to leave the heterologous enzymes in the plant biomass through the pretreatment and utilize them directly during enzymatic hydrolysis. However, as most thermochemical pretreatments typically operate at upwards of 100 °C (Hu *et al*., 2012), enzymes with high optimum temperatures (i.e. hyperthermal or thermotolerant enzymes) are needed in order to retain enzymatic activity following processing. Two hyperthermal endoglucanases that have been studied are *A. cellulolyticus* AcCel5A (E1) and *Sulfolobus solfataricus* SsCel12 (SSO1354), both of which have optimum temperatures of ~80–90 °C (Huang *et al*., 2005; Klose *et al*., 2012; Tucker *et al*., 1989). However, while thermal stability is important, pH stability of these enzymes and the interaction between pH and temperature are also important factors to consider (Sun *et al*., 2007; Verma *et al*., 2010). The optimum pH for AcCel5A (E1) is ~5.0 (Dai *et al*., 2000) and *S. solfataricus* SsCel12 (SSO1354) is ~4.5 (Klose *et al*., 2012), and extractions or pretreatments of the biomass that operate far from these values will likely lead to a loss of enzyme activity, even at temperatures below 80 °C. This is likely the reason for the drop AcCel5A (E1) activity (35% of the untreated) following low‐severity AFEX pretreatment (60 °C, pH > 8) of transgenic tobacco (Teymouri *et al*., 2004), and the complete loss of AcCel5A activity for dilute acid pretreated (110–170 °C; pH < 3) transgenic corn stover (Brunecky *et al*., 2011). For ionic liquid pretreatments, where the pretreatment solvent consists of an organic cation coupled with an organic or inorganic cation and acts by solvating a portion of the biomass (either polymeric sugars and/or lignin) (Mora‐Pale *et al*., 2011), salt tolerance of the enzymes may also be an issue that needs to be taken into consideration (Klose *et al*., 2012). Work has already been done to heterologously express alkali‐tolerant thermophilic enzymes (optimum pH = 8–9, optimum temp. = 60–70 °C) (Hu and Ragauskas, 2012; Leelavathi *et al*., 2003) and acid‐tolerant thermophilic enzymes (pH range = 1–4, optimum temp. = 60–70 °C) *in planta* (Xu *et al*., 2013; Zhang *et al*., 2013). It is likely that thermotolerant enzymes from acidophiles, alkaliphiles or halophiles that are more tolerant of pH extremes and ionic strength would better handle the extreme pretreatment conditions needed for deconstruction of the plant cell wall. However given the diversity of pretreatment methods, the properties of the enzymes would need to be matched to the chosen pretreatment system.

### Industrial activities towards the production and use of plant‐generated enzymes

In 2011, Syngenta began producing and distributing a corn hybrid, Enogen^®^, that produces a heterologous α‐amylase in its seeds, eliminating the need to add α‐amylase during the dry grind corn ethanol process (Hood and Requesens, 2012). Syngenta has also developed the Cellerate^™^ technology to simultaneously convert corn starch and fibre, the lignocellulosic portion of corn grain, to ethanol (Lundy *et al*., 2015). In the future, they may expand to include other heterologous lignocellulolytic enzymes (Lebel *et al*., 2008, 2010, 2013; Miles, 2012a,b). Agrivida is also attempting to heterologously express cellulases and hemicellulases in maize seeds. Their key technology centres around the use of bacterial inteins that are added internal to the enzyme and restrict activity while the plant is growing, but self‐splice upon exposure to elevated temperatures during pretreatment (>59 °C) (Gray *et al*., 2011b; Shen *et al*., 2012). Using this technology, inteins were inserted into an endoxylanase (XynB), which resulted in higher glucose and xylose yields compared with the wild type (Shen *et al*., 2012). Xylanase activity was also retained following mild ammonium bisulfite pretreatment, with reduced need for external xylanase supplementation during hydrolysis (Zhang *et al*., 2011). Their heterologously produced endoglucanase was also able to completely replace EG in a synthetic enzyme cocktail (Zhang *et al*., 2011). Agrivida is also working on expressing multiple enzymes within the same plant and so far has successfully stacked two to three enzymes (Zhang *et al*., 2011).

## Conclusions

A great deal of progress has been made in the past decade on the heterologous production of cell wall‐degrading enzymes *in planta*. Research has moved beyond the much‐studied endoglucanase, E1 (AcCel5A), to the expression of many different types of enzymes from a variety of microbial sources (Tables 1 and S2). Using a combination of genetic tools and strategies, recombinant enzymes have also been accumulated at levels as high as 50% total soluble protein (TSP) (Hahn *et al*., 2014) without compromising plant growth and biomass yields. It is expected that in the next decade, the heterologous enzyme yield *in planta* could consistently reach these levels or higher using a number of methods. Subcellular targeting in particular has been demonstrated as a successful strategy to both sequester large quantities of heterologous cell wall‐deconstructing enzymes away from plant cell walls, preventing possible deterioration, while limiting access to and degradation by cellular proteases. Other strategies may include using high biomass producing varieties for the host plant, targeting the chloroplast via homologous recombination and manipulation of DB regions to enhance expression, utilization of optimized regulatory sequences, optimizing promoter enhancers for both the enzyme and subcellular compartment, targeting multiple subcellular organelles for enzyme containment, stacking enzyme genes or utilizing alternative splicing for production of multiple enzymes within a single construct, and producing chimeric enzymes with multiple activities. Conventional breeding could also be investigated further as a means to achieve some of these goals. From an application standpoint, greater focus needs to be directed towards retention of CBMs in enzymes that require them for proper functionality, maintenance of enzyme activity during storage and heterologous production of enzymes that are tolerant to the high temperatures and extreme pH conditions found during conventional thermochemical pretreatment methods. Development of a sustainable enzyme production platform is still underway. However, successful production of cell wall‐deconstructing enzymes within bioenergy crops holds strong potential for helping to establish sustainable and profitable bioenergy production systems.

## Supporting information


**Figure S1** Cellulose deconstructing and mixed‐linkage glucan‐degrading enzyme activities.
**Figure S2** Xyloglucan‐ and glucuronoarabinoxylan‐degrading enzyme activities.
**Figure S3** Galactoglucomannan‐ and galacturonan‐degrading enzyme activities.
**Figure S4** Rhamnogalacturonan I‐ and II‐degrading enzyme activities.
**Table S1** Full summary of subcellular targeting of cell wall‐degrading enzymes since 2005: arranged by subcellular compartment.
**Table S2** Summary of heterologous production of cell wall‐degrading enzymes since 2005: arranged by enzyme.
**Appendix S1** Microbial cell wall‐deconstructing enzyme classification and mode of action.
