# Supplementary material for: Strategies for the production of cell wall‐deconstructing enzymes in lignocellulosic biomass and their utilization for biofuel production
Source: Plant Biotechnol J. 2015 Dec 2;14(6):1329–44. doi: 10.1111/pbi.12505 (PMC5063159; doi:10.1111/pbi.12505)
Supplement: Supplementary file 1 — Figure S1 Cellulose deconstructing and mixed‐linkage glucan‐degrading enzyme activities. Figure S2 Xyloglucan‐ and glucuronoarabinoxylan‐degrading enzyme activities. Figure S3 Galactoglucomannan‐ and galacturonan‐degrading enzyme activities. Figure S4 Rhamnogalacturonan I‐ and II‐degrading enzyme activities. Table S1 Full summary of subcellular targeting of cell wall‐degrading enzymes since 2005: arranged by subcellular compartment. Table S2 Summary of heterologous production of cell wall‐degrading enzymes since 2005: arranged by enzyme. Appendix S1 Microbial cell wall‐deconstructing enzyme classification and mode of action. [file PBI-14-1329-s001.pdf]

Strategies for the Production of Cell Wall-Deconstructing Enzymes in Lignocellulosic Biomass and Their Utilization for Biofuel Production

Sang-Hyuck Park[a]*, Rebecca Garlock Ong[bc] and Mariam Sticklen[a]

[a]Department of Plant, Soil and Microbial Sciences, Michigan State University, East Lansing, MI 48824; [b]Department of Chemical Engineering and Materials Science, DOE Great Lakes Bioenergy Research Center, Michigan State University, East Lansing MI 48824; [c]Department of Chemical Engineering, Michigan Technological University, Houghton, MI 49931

*Corresponding author (current address): Sang-Hyuck Park, Genomics and Bioinformatics Research Unit, USDA-ARS, 141 Experiment Station Rd., Stoneville, MS 38776, United States. Email:sang.park@ars.usda.gov

Supplemental Info

Contents

MICROBIAL CELL WALL-DECONSTRUCTING ENZYME CLASSIFICATION AND MODE OF ACTION.. 2

Figure S1: Cellulose deconstructing and mixed-linkage glucan-degrading enzyme activities. 6

Figure S2: Xyloglucan- and glucuronoarabinoxylan-degrading enzyme activities. 7

Figure S3: Galactoglucomannan and galacturonan degrading enzyme activities. 8

Figure S4: Rhamnogalacturonan I and II degrading enzyme activities. 9

Table S1: Full summary of subcellular targeting of cell wall degrading enzymes since 2005: Arranged by subcellular compartment... 10

Table S2: Summary of heterologous production of cell wall degrading enzymes since 2005: Arranged by enzyme. 24

References... 37

MICROBIAL CELL WALL-DECONSTRUCTING ENZYME CLASSIFICATION AND MODE OF ACTION

Cell wall-deconstructing enzymes are broadly classified based on their modes of action. The Carbohydrate-Active EnZymes (CAZy) database classifies enzymes that catalyze the assembly and deconstruction of complex carbohydrates and phenolics (Lombard et al., 2014). Within the database, the cell wall-deconstructing enzymes are subdivided into different groups based on the mode of action of their catalytic domains. These include: (1) *glycoside hydrolases* (GH: EC 3.2.1.-) that act by hydrolytically cleaving or rearranging bonds between their carbohydrates; (2) *polysaccharide lyases* (PL: EC 4.2.2.-) that cleave the bonds between uronic acid residues; (3) *carbohydrate esterases* (CE) that cleave the ester groups from polysaccharides (e.g. acetyl, methyl, and feruloyl esters); and (4) enzymes with *auxiliary activities* (AA) or redox enzymes, including ligninolytic enzymes (laccases and peroxidases) and lytic polysaccharide monooxygenases (LPMOs) (Gilbert, 2010; Lombard et al., 2014; van den Brink and de Vries, 2011). In addition to the above, many enzymes are connected to a carbohydrate-binding module (CBM) via a flexible linker that assists in binding and movement of the catalytic domain along the polysaccharide chain (Black et al., 1996; Gilbert et al., 2013; Hägglund et al., 2003; Lombard et al., 2010; Shallom and Shoham, 2003).

While many cell wall-deconstructing enzymes are active on only one substrate, multi-functional enzymes or “chimaeras,” possess more than one enzymatic activity and can be either naturally occurring or synthetically produced. These enzymes have multiple activities either because they possess an active site that is able to accommodate more than one substrate, or they have multiple active sites, each of which has activity toward a different substrate (Cho et al., 2006; Elleuche, 2015; Fan and Yuan, 2010; Ferrer et al., 2012).

Glycoside Hydrolases (GHs; EC 3.2.1.-)

Cellulases: The cellulases are a group of glycoside hydrolases that are required to break down cellulose microfibrils **(Figure S1)**. At least three cellulase enzymes are necessary to function synergistically in order to effectively degrade cellulose. These include: cellulase (endo-1,4- β-glucanase: EC 3.2.1.4), cellulose-1,4- β-cellobiosidase (EC 3.2.1.91 or EC 3.2.1.176), and β-glucosidase (EC 3.2.1.21). Cellulase, commonly called endoglucanase (EG) acts by generating new reducing and non-reducing ends within the cellulose chains by randomly cleaving β-1,4-glucosidic linkages (Park et al., 2011). Cellobiohydrolases, sometimes referred to as exoglucanases because they attack the ends of the glucan

chain, act either on the reducing end (CBHI: EC 3.2.1.176) or non-reducing end (CBHII: EC 3.2.1.91) of the chain, cleaving off cellobiose (dimeric β-1,4-linked glucose) units (Nutt et al., 1998). β-glucosidases convert soluble cellobiose to glucose. In addition to the three necessary enzyme activities, enzymes with glucan 1,4- β-glucosidase activity can also act on cellulose, releasing monomeric glucose from the reducing end of the glucan chain.

Hemicellulases and Pectinases: The hemicelluloses and pectins are two classes of single-chain, amorphous polysaccharides, substituted with various side chains. These include monosaccharides, uronic acids, acetyl groups, and/or complex branches comprised of multiple residues (Mohnen, 2008; Scheller and Ulvskov, 2010). The main structural difference between the two classes is in the structure of the polymer backbone. Hemicelluloses are characterized by the presence of β-1,4-linkages between backbone sugars, and are divided into four main classes based on their backbone polymers: mixed-linkage glucan (MLG), xyloglucan, glucuronoarabinoxylan, and galactoglucomannan, In contrast, pectins have either a homogalacturonan (HG) backbone comprised of α-1,4-linked galacturonic acid, or a rhamnogalacturonan (RG) backbone, which is a disaccharide repeat of [-α-Galacturonic Acid-1,2- α-Rhamnose-1,4-] (Mohnen, 2008). All hemicelluloses and pectins require endo-acting enzymes that are specific for their respective polymeric backbones (**Figures S1-S4)**.

Because of the complexity and variety of linkages present in hemicelluloses and pectins, there are a large number of additional enzymes that are necessary to degrade them (Bonnin et al., 2014; Culleton et al., 2013; Shallom and Shoham, 2003). Almost all of the pectins and hemicelluloses have side-chains, and those of RG I and RG II, in particular, are extremely complex (**Figure S4**). Generally these side-chains need to be removed before endo-acting enzymes are able to effectively break down the backbone, although in some cases the presence of side-chains may be necessary for recognition of substrates by certain enzymes (Hurlbert and Preston, 2001). Complete removal of side-chains can require an extremely large number of enzyme activities, as a different enzyme activity is required for each unique type of sugar and linkage position (**Figure S2-S4**). But even when present as a small proportion of the enzyme mixture, the addition of side-chain removing enzymes can significantly improve enzymatic hydrolysis yields (Banerjee et al., 2010; Gao et al., 2011; Jabbour et al., 2014).

Polysaccharide Lyases (PLs; EC 4.2.2.-**)**

Polysaccharide lyases cleave the bond between a uronic acid or ester moiety without the use of water and leave a hexenuronic acid residue or ester as the new reducing end

(Garron and Cygler, 2010; Lombard et al., 2010). Most of the PLs that have been characterized have activity on pectin backbones. Pectin lyases (EC 4.2.2.10) typically act on highly methylated HG, pectate lyases (4.2.2.2) act on non-methylated HG, and RG lyases (EC 4.2.2.24) act on the RG backbone (Bonnin et al., 2014). Many of the PLs are activated by bivalent catalytic metals, such as Ca^{2+}, Mg^{2+}, or Mn^{2+} (Abbott et al., 2013; Cordula et al., 2014; Gibson, 2012).

Carbohydrate Esterases (CEs)

There are a number of compounds that are ester-linked to pectin and hemicellulose, including acetyl and methyl groups, and phenolic acids. Most of the pectins and hemicelluloses are O-acetyl esterified at various positions on the sugar backbone (Biely, 2012; Pawar et al., 2013). Uronic acid side-chains, such as glucuronic acid on dicot xylans and galacturonic acid on homogalacturonan can also be methyl-esterified (Bonnin et al., 2014; Scheller and Ulvskov, 2010). The frequency of acetylation and methylation impact carbohydrate interactions and cell wall properties, and their presence sterically hinders the binding of glycosyl hydrolyases (Biely, 2012). Of the plant phenolic esters, ferulic acid is the only one that has been shown to be ester-linked to structural carbohydrates within the plant cell wall. Grasses in particular are characterized by ferulic acid residues that are ester-linked to the arabinose side-chains of glucuronoarabinoxylan, and cross-link to lignin within the cell wall (Hatfield et al., 1999). Ferulic acid can also ester-link to arabinose and galactose residues in pectin (Bonnin et al., 2014; Koseki et al., 2009). Because of CE specificity, a variety of activities are necessary for removal of all types of esters, including: non-specific acetyl esterases (AEs: EC 3.1.1.6), acetylxylan esterases (AXEs: EC 3.1.1.72), feruloyl esterases (EC 3.1.1.73), acetyl glucomannan esterases (AGMEs: EC.3.1.1.-), pectin methyl esterases (EC 3.1.1.11), pectin acetyl esterases (EC 3.1.1.-), and rhamnogalacturonan acetyl esterase (EC 3.1.1.86) that is specific to RG I (Bonnin et al., 2014; Pawar et al., 2013).

Enzymes with Auxiliary Activities (AAs)

In 2013, the “Auxiliary Activities” group was added to the CAZy database in order to encompass the families of redox enzymes, including lignin-active enzymes such as peroxidases and laccases, and the newly characterized lytic polysaccharide monoxygenases (LPMOs) (Levasseur et al., 2013). The LPMOs were originally classified as glycosyl hydrolases, but have recently been discovered to be copper-dependent enzymes that oxidatively cleave carbohydrates, including cellulose, chitin, and hemicelluloses (Agger et al., 2014; Levasseur et al., 2013).

Lignin is a phenylpropanoid polymer and a major constituent of plant secondary cell walls, and cross-links to the other cell wall components, limiting the accessibility of cell wall-degrading enzymes. A number of enzymes are known to target lignin, and the most well known are lignin peroxidase, manganese peroxidase, and laccase (Janusz et al., 2013). Lignin peroxidases (E.C.1.11.1.14), manganese peroxidases (EC 1.11.1.13), and versatile peroxidases (EC 1.11.1.16) are heme-containing enzymes that catalyze oxidative degradation of lignin (Cohen et al., 2009; Feng et al., 2011; Hofrichter, 2002; Liers et al., 2011; Lundell et al., 2010; Wong, 2009). Laccases (EC 1.10.3.2) are multi-copper oxidases that are believed to be involved in the polymerization and depolymerization of lignin (Bugg et al., 2011; Liers et al., 2011), though they may not be essential for lignin decomposition (Lundell et al., 2010).

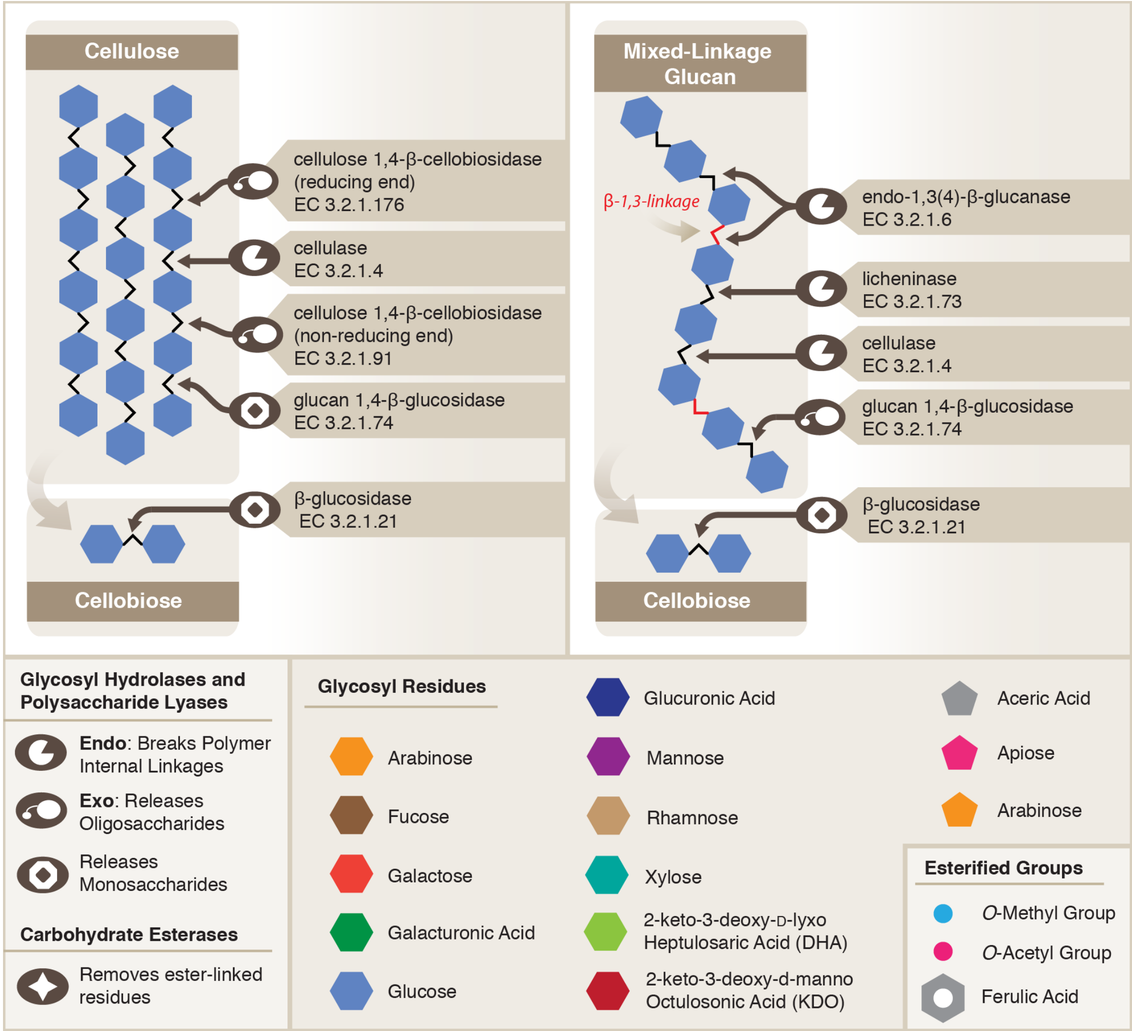

Figure S1: Cellulose deconstructing and mixed-linkage glucan-degrading enzyme activities. β-1,3-linkages between glucose residues are shown in red and the reducing end of the polysaccharide is at the top of the figure.

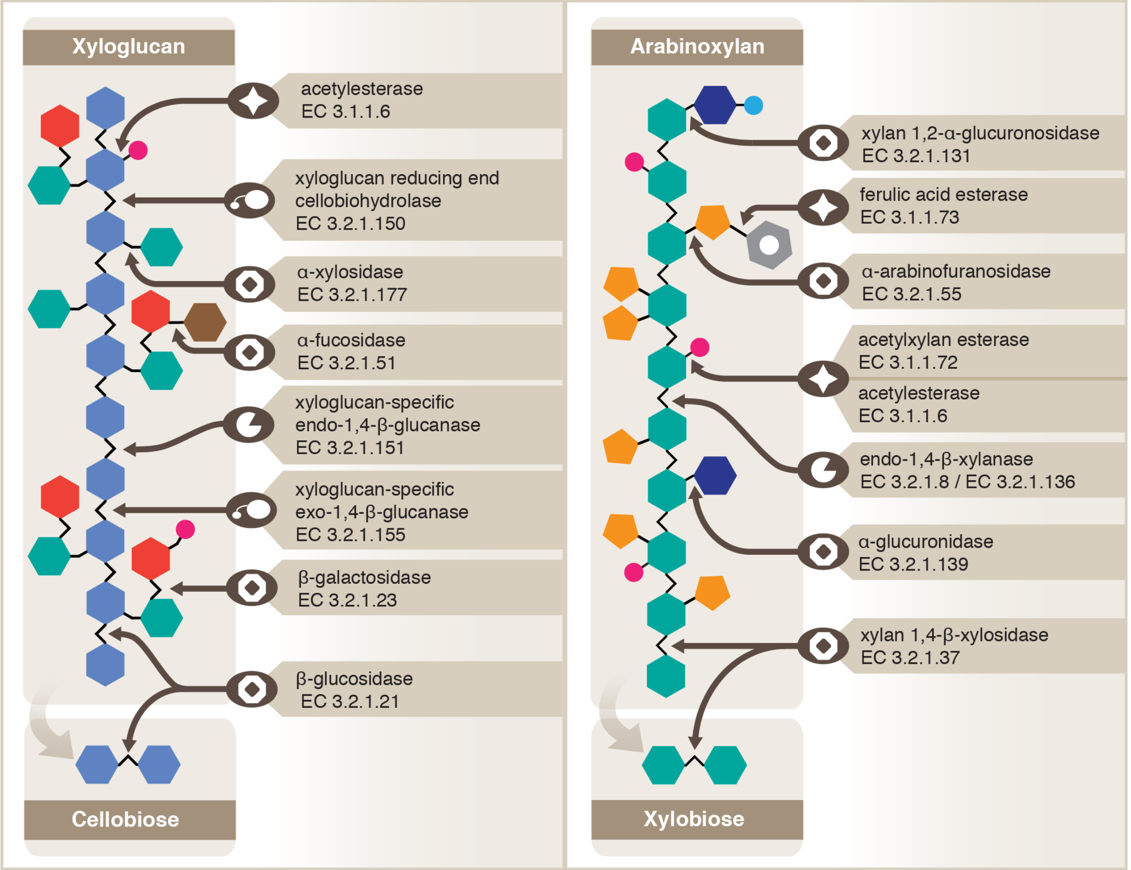

Figure S2: Xyloglucan- and glucuronoarabinoxylan-degrading enzyme activities.
The reducing end of the polysaccharide is at the top of the figure. Please refer to Figure S1 for the legend.

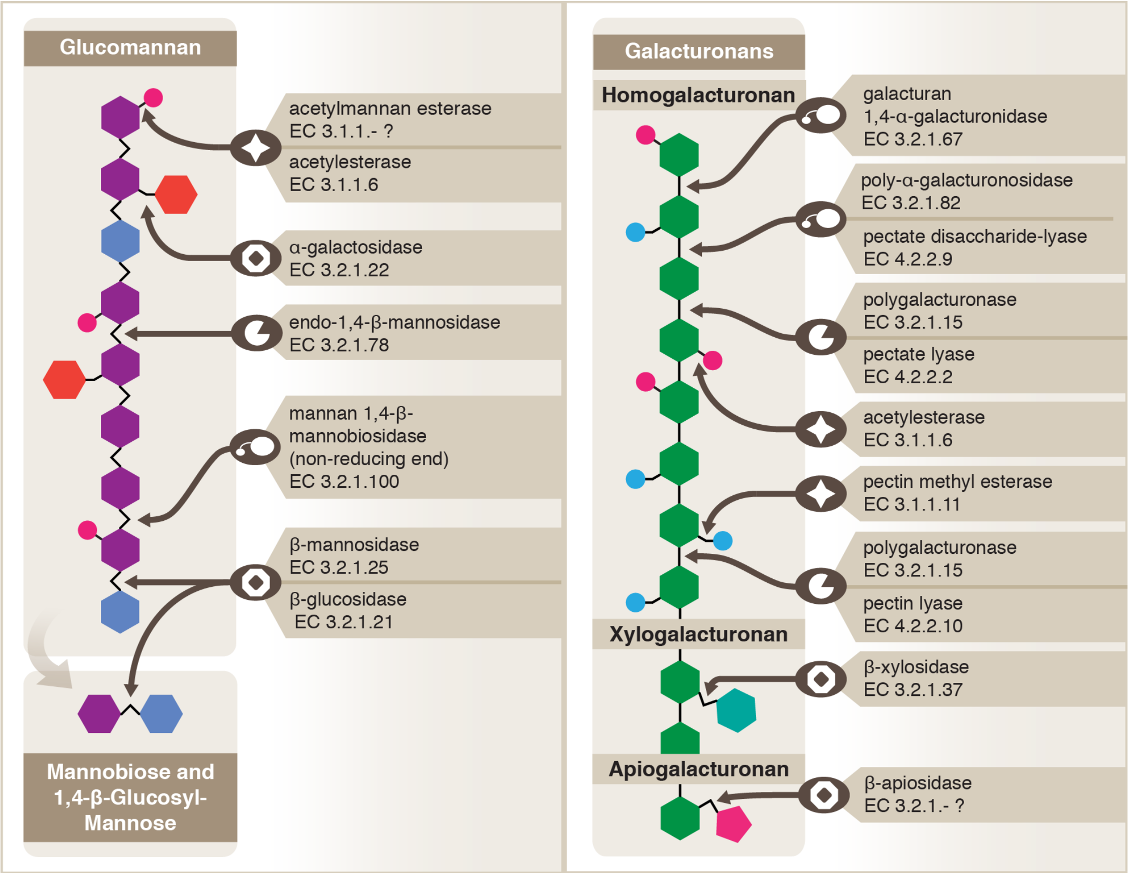

Figure S3: Galactoglucomannan and galacturonan degrading enzyme activities.
Pectic galacturonans can be unsubstituted (with only acetic and methyl esters) as in the case of homogalacturonan, or with sugar side-chains as is the case for xylogalacturonan (xylose) and apiogalacturonan (apiose). The reducing end of the polysaccharide is at the top of the figure. Please refer to Figure S1 for the legend.

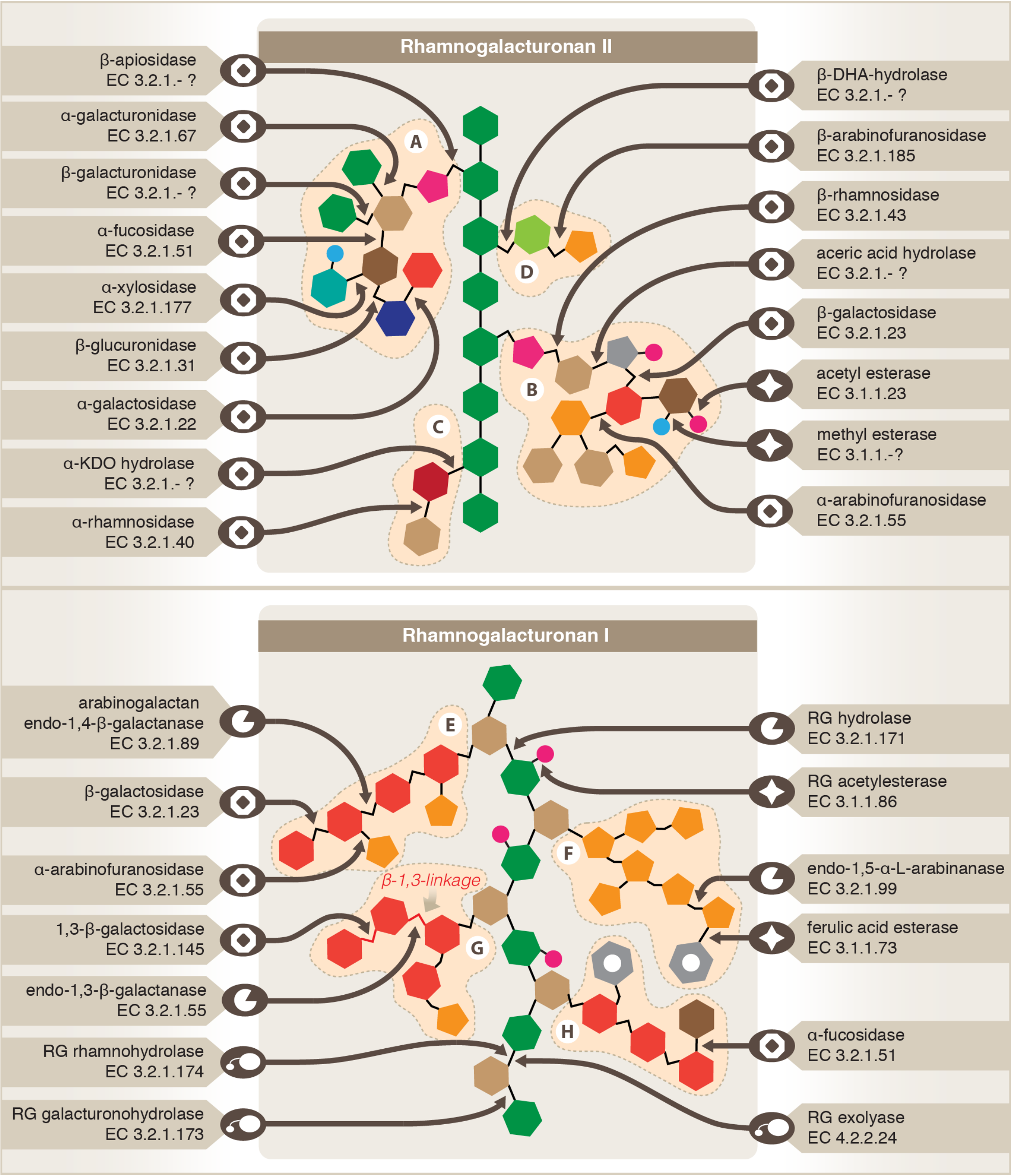

Figure S4: Rhamnogalacturonan I and II degrading enzyme activities.
The enzyme activities required to degrade the RG II backbone, are the same as those needed for homogalacturonan, shown in Figure 3. The conserved side branches in RG II are labeled as (A-D) in accordance with convention. In contrast, RG I side chains are highly variable and as pictured are simply a representation of the general structure. RG I side chains are labeled as follows: E) type I arabinogalactan, F) arabinan, G) type II arabinogalactan, and H) galactan. The reducing end of the polysaccharide is at the top of the figure. Please refer to Figure S1 for the legend.

Table S1: Full summary of subcellular targeting of cell wall degrading enzymes since 2005: Arranged by subcellular compartment.

Abbreviations: ***alc*****Amin35S**: alcohol inducible promoter based on CaMV 35S; **ALE**: barley aleurain vacuole targeting signal; **aps**: amplification promoting sequence; **BAASS**: Barley α-amylase signal sequence; **CAB:** Chlorophyll *a/b* binding protein; **CALSP:** tobacco calreticulin signal peptide; **CaMV35SS**: double CaMV 35S promoter; **Cox IV**: Yeast cytochrome c oxidase subunit; **CTP:** artificial dicot chloroplast targeting sequence; **CTPP**: C-terminal propeptide tobacco chitinase vacuolar sorting signal; **DELKAEAK**: vacuole sorting determinant; **FNR:** ferredoxin-NADP$^+$-oxidoreductase; **Frameshift KDEL**: frameshifted terminal peptide (ETTEG) that removes ER retention; **Glb1:** Maize globulin1; **Glb2**: globulin2; **Glub-4:** rice glutelin B-4 gene; **Gt1**: rice glutelin Gt1 promoter; **(SE/DI/L)KDEL/HDEL**: endoplasmic reticulum retention signal; **LmSee1**: Lolium multiflorium senescence enhanced gene promoter; **Mac**: hybrid of Ti plasmid mannopine synthetase promoter and cauliflower mosaic virus 35S promoter enhancer; **MMA:** leader peptide derived from murine monoclonal antibody mAb24; **MMV**: Mirabilis mosaic virus promoter; **MRbcSK-1A**: three alfalfa RbcS promoters (RbcSK-1A) without negative regulatory region; **OlexA-46**: β-estradiol inducible promoter; **Pact2:** Arabidopsis actin 2 promoter; **PepC**: phosphoenolpyruvate carboxylase; **PpsbA:** PSII protein D1 promoter; **PPI**: Potato protease inhibitor II; **Prrn**: Tobacco 16S ribosomal ERNA promoter; **PR-S/PR1a/PR1b**: pathogenesis-related proteins; **PvPGIP1:** *P. vulgaris* polygalacturonase-inhibiting protein; **RA**: Rubisco activase; **RbcS**: Rubisco small subunit; **Rice SGR**: Rice Stay Green gene; **RST**: rat sialyl transferase Golgi targeting motif; **Rubi3**: rice ubiquitin promoter; **SAG12:** Arabidopsis senescence inducible promoter; **SKL**: peroxisome targeting C-terminal sequence; **SPER**: endoplasmic reticulum targeting signal peptide; **T7g10**: bacteriophage T7 gene 10 N-terminal enhancer; **VT:** vacuole targeting signal peptide; **Zm-leg1A**: maize legumin promoter

Signal Peptide / Terminator Sequence	Enzyme Activity	Enzyme Name (Traditional/ Family)	Enzyme Source Organism	Promoter	Plant Species and Tissue	% Total Soluble Protein	Ref.
**Cytosol**							
-	Endo-1,4-β-glucanase (3.2.1.4)	E1/AcCel5A	*Acidothermus cellulolyticus*	Mac	Tobacco Leaves	-	(Dai et al., 2005)
				CaMV 35S	Duckweed Plants	0.24%	(Sun et al., 2007)
				Gt1	Rice Seeds	-	(Apgar et al., 2012)
		ENG1/-	*Oryza sativa*	Maize Ubiquitin	Rice Leaves	-	(Nigorikawa et al., 2012)
		E2/TfCel6A	*Thermobifida fusca*	CaMV35SS	Tobacco Leaves	-	(Jiang et al., 2011)
		TmCel5A	*Thermotoga maritima*	RbcSK-1A	Tobacco Leaves	0%	(Mahadevan et al., 2011)
				Pact2	Tobacco Leaves	12.5%	(Hahn et al., 2014)

Signal Peptide / Terminator Sequence	Enzyme Activity	Enzyme Name (Traditional/ Family)	Enzyme Source Organism	Promoter	Plant Species and Tissue	% Total Soluble Protein	Ref.
**Cytosol (cont.)**							
-	Cellulose 1,4-β-cellobiosidase[NR] (3.2.1.91)	EXG1/-	*Oryza sativa*	Maize Ubiquitin	Rice Leaves	-	(Nigorikawa et al., 2012)
				Rice SGR	Rice Straw	-	(Furukawa et al., 2014)
		E3/TfCel6B	*Thermobifida fusca*	CaMV35SS	Tobacco Leaves	-	(Jiang et al., 2011)
	1,4-β-glucosidase (3.2.1.21)	HgBgl4	*Humicola grisea*	Pact2	Tobacco Leaves	50%	(Hahn et al., 2014)
		BEG1/-	*Oryza sativa*	Rice actin	Rice Leaves	-	(Nigorikawa et al., 2012)
		BglB/TmBgl3	*Thermotoga maritima*	RbcSK-1A	Tobacco Leaves	4.5%	(Jung et al., 2010; Jung et al., 2013)
				MRbcSK-1A + aps	Tobacco Leaves	8.3%	(Jung et al., 2013)
	Endo-1,4-β-xylanase (3.2.1.8)	XynB/CsXyn10B	*Clostridium stercorarium*	CaMV 35S	Tobacco Leaves	0.1%	(Kimura et al., 2010)
				Rice actin act1	Rice Leaves	0.1 – 0.2%	(Kimura et al., 2010)
		XynB/SoXyn11	*Streptomyces olivaceoviridis* A1	CaMV 35SS	Potato Leaves and Tubers	5%	(Yang et al., 2007)
		XylII/TrXyn11A	*Trichoderma reesei*[c]	RbcSK-1A	Arabidopsis Leaves	1.2%	(Bae et al., 2006)
				CaMV 35S	Arabidopsis Leaves	1.4%	(Bae et al., 2008)
		ATX/Xyn11	Synthetic Construct	CaMV 35S	Rice Leaves	-	(Weng et al., 2013)
	Polygalacturonase (3.2.1.15)	AnPgaI	*Aspergillus niger*	CaMV 35SS	Tobacco Leaves	0%	(Pereira et al., 2014)
	4-O-methyl-glucuronoyl methylesterase (3.1.1.-)	PcGCE	*Phanerochaete carnosa*	CaMV 35S	Arabidopsis Stems and Leaves	-	(Tsai et al., 2012)
	Laccase (1.10.3.2)	Cvl3 + CBM	*Trametes versicolor*	Ubiquitin	Rice Culm	-	(Furukawa et al., 2013)

Signal Peptide / Terminator Sequence	Enzyme Activity	Enzyme Name (Traditional/ Family)	Enzyme Source Organism	Promoter	Plant Species and Tissue	% Total Soluble Protein	Ref.
-	Endo-1,4-β-xylanase / Feruloyl esterase[a] (3.2.1.8 / 3.1.1.73)	XynZ/RtXyn10	*Ruminiclostridium thermocellum*	MMV	Tobacco Leaves	1.5 – 3.1%	(Chatterjee et al., 2010)
	Chimeric: Endo-1,4-β-xylanase / Feruloyl esterase / α-arabinofuranosidase[a] (3.2.1.8 / 3.1.1.73 / 3.2.1.55)	XynZ/RtXyn10; AbfA/GsAbf51	*Ruminiclostridium thermocellum (Xyn/Fae); Geobacillus stearothermophilus (Abf)*	MMV	Tobacco Leaves	0.6 – 2.0%	(Fan and Yuan, 2010)
**Apoplast**							
Mutated ALE	Feruloyl esterase (3.1.1.73)	AnFaeA	*Aspergillus niger*	Rice Actin 1	Tall Fescue Leaves	-	(Buanafina et al., 2010)
				Soybean Heat Shock	Tall Fescue Leaves	-	(Buanafina et al., 2010)
Mutated ALE / Frameshift KDEL	Feruloyl esterase (3.1.1.73)	AnFaeA	*Aspergillus niger*	LmSee1	Tall Fescue Leaves	-	(Buanafina et al., 2010)
Arabidopsis 2S2	Endo-1,4-β-xylanase / Feruloyl esterase[a] (3.2.1.8 / 3.1.1.73)	XynZ/RtXyn10	*Ruminiclostridium thermocellum*	MMV	Tobacco Leaves	2.2 – 4.1%	(Chatterjee et al., 2010)
Arabidopsis β - expansin	Acetylxylan esterase (3.1.1.72)	AnAXE	*Aspergillus nidulans*	CaMV 35S	Arabidopsis Leaves & Stems	-	(Pogorelko et al., 2011; Pogorelko et al., 2013)
	RG acetyl esterase (3.1.1.86)	AnRAE	*Aspergillus nidulans*	CaMV 35S	Arabidopsis Leaves	-	(Pogorelko et al., 2013)
	Feruloyl esterase (3.1.1.73)	AnFAE	*Aspergillus nidulans*	CaMV 35S	Arabidopsis Leaves & Stems	-	(Pogorelko et al., 2011)
BAASS	Endo-1,4-β-glucanase (3.2.1.4)	E1/AcCel5A	*Acidothermus cellulolyticus*	Glb1	Maize Seeds	ND	(Hood et al., 2007)
				Glb1	Maize Seeds	7.1%	(Egelkrout et al., 2013)
				Glb2 + Glb1 + pr26	Maize Seeds	18.8%	(Egelkrout et al., 2013)

Signal Peptide / Terminator Sequence	Enzyme Activity	Enzyme Name (Traditional/ Family)	Enzyme Source Organism	Promoter	Plant Species and Tissue	% Total Soluble Protein	Ref.
**Apoplast (cont.)**							
BAASS	Cellulose 1,4-β-cellobiosidase[R] (3.2.1.176)	CBHI/HgCel7A	*Humicola grisea*	Pact2	Tobacco Leaves	10%	(Hahn et al., 2014)
		CBHI/TrCel7A	*Trichoderma reesei*[c]	Glb1	Maize Seeds	17.8%	(Hood et al., 2007)
				Glb1	Maize Seeds	3.2%	(Egelkrout et al., 2013)
				Glb1 + pr36 + Glb2	Maize Seeds	4.1%	(Egelkrout et al., 2013)
				Pact2	Tobacco Leaves	12.5%	(Hahn et al., 2014)
	Cellulose 1,4-β-cellobiosidase[NR] (3.2.1.91)	CBHII/TrCel6A	*Trichoderma reesei*[c]	Rice Glutelin	Maize Seeds	30%	(Devaiah et al., 2013)
	Endo-1,4-β-xylanase (3.2.1.8)	BSX/Xyn10	*Bacillus* sp. NG-27	Rubi3	Maize Stover	0.1%	(Gray et al., 2011a)
		XynA/BsXyn11	*Bacillus subtilis*	Wheat Glutenin 1DX5	Wheat Seeds	-	(Harholt et al., 2010)
		XynB/CsXyn10B	*Clostridium stercorarium*	Rubi3	Maize Stover	0.1%	(Gray et al., 2011a)
		XynA/DtXyn10	*Dictyoglomus thermophilum*	CaMV 35S	Arabidopsis Leaves and Stems	14%	(Borkhardt et al., 2010)
		XynB/DtXyn11	*Dictyoglomus thermophilum*	CaMV 35S	Arabidopsis Leaves and Stems	3%	(Borkhardt et al., 2010)
				Rubi3	Maize Seeds	-	(Shen et al., 2012)
	Feruloyl esterase (3.1.1.73)	FaeA	*Aspergillus niger*	Wheat Glutenin 1DX5	Wheat Seeds	-	(Harholt et al., 2010)
	Manganese peroxidase (1.11.1.13)	MnP2	*Phanerochaete chrysosporium*	Glb1	Maize Seeds	6-14%	(Clough et al., 2006)

Signal Peptide / Terminator Sequence	Enzyme Activity	Enzyme Name (Traditional/ Family)	Enzyme Source Organism	Promoter	Plant Species and Tissue	% Total Soluble Protein	Ref.
**Apoplast (cont.)**							
CALSP	Endo-1,4-β-glucanase (3.2.1.4)	E2/TfCel6A	*Thermobifida fusca*	CaMV 35SS	Tobacco Leaves	-	(Jiang et al., 2011)
	Cellulose 1,4-β-cellobiosidase[NR] (3.2.1.91)	E3/TfCel6B	*Thermobifida fusca*	CaMV 35SS	Tobacco Leaves	-	(Jiang et al., 2011)
Chitinase 1	Endo-1,4-β-mannosidase (3.2.1.78)	Amn5A/Man5A	*Bacillus* sp. JAMB-602	CaMV 35S	Tobacco Leaves	-	(Hoshikawa et al., 2012)
Maize expansin B	Acetyl xylan esterase (3.1.1.72)	AnAXE	*Aspergillus nidulans*	Maize Ubiquitin	Brachypodium Leaves	-	(Pogorelko et al., 2013)
	Rhamnogalacturonan acetyl esterase (3.1.1.86)	AnRAE	*Aspergillus nidulans*	Maize Ubiquitin	Brachypodium Leaves	-	(Pogorelko et al., 2013)
MMA	Endo-1,4-β-glucanase (3.2.1.4)	SsCel12	*Sulfolobus solfataricus*	CaMV 35SS	Tobacco	-	(Klose et al., 2012)
		TrCel5A	*Trichoderma reesei*[c]	CaMV 35SS	Tobacco Leaves	-	(Klose et al., 2013)
				*alc*Amin35S	Tobacco Leaves	-	(Klose et al., 2013)
				CaMV 35SS	Tobacco Leaves	-	(Klose et al., 2015)
PPI	Endo-1,4-β-xylanase (3.2.1.8)	XynB/SoXyn11	*Streptomyces olivaceoviridis* A1	CaMV 35SS	Potato Leaves and Tubers	-	(Yang et al., 2007)
		XynII/TrXyn11A	*Trichoderma reesei*[c]	Rice Actin 1	Tall Fescue Leaves	-	(Buanafina et al., 2012)
				LmSee1	Tall Fescue Leaves	-	(Buanafina et al., 2015; Buanafina et al., 2012)
	Feruloyl esterase (3.1.1.73)	FaeA	*Aspergillus niger*	Rice Actin 1	Tall Fescue Leaves	-	(Buanafina et al., 2015; Buanafina et al., 2010)

Signal Peptide / Terminator Sequence	Enzyme Activity	Enzyme Name (Traditional/ Family)	Enzyme Source Organism	Promoter	Plant Species and Tissue	% Total Soluble Protein	Ref.
**Apoplast (cont.)**							
Pr1a / Pr1b / PR-S	Endo-1,4-β-glucanase (3.2.1.4)	E1/AcCel5A	*Acidothermus cellulolyticus*	Mac	Tobacco Leaves	-	(Dai et al., 2005)
				CaMV 35S	Maize Leaves and Roots	2.1%	(Biswas et al., 2006)
				CaMV 35S	Maize Leaves	1.2%	(Ransom et al., 2007)
				CaMV 35S	Rice Leaves	4.9%	(Oraby et al., 2007)
				Mac	Rice Leaves	6.1%	(Chou et al., 2011)
		TmCel5A	*Thermotoga maritima*	Alfalfa RbcSK-1A	Tobacco Leaves	1.2%	(Mahadevan et al., 2011)
	Cellulose 1,4-β-cellobiosidase[R] (3.2.1.176)	CBHI/TrCel7A	*Trichoderma reesei*[c]	CaMV 35S	Maize and Tobacco Leaves	-	(Park et al., 2011)
	Polygalacturonase (3.2.1.15)	AnPgaI	*Aspergillus niger*	CaMV 35SS	Tobacco Leaves	2.5%	(Pereira et al., 2014)
	Feruloyl esterase (3.1.1.73)	AnFaeB	*Aspergillus niger*	tCUP4	Alfalfa Leaves	-	(Badhan et al., 2014)
PttCel9B3	4-O-methyl-glucuronoyl methylesterase (3.1.1.-)	PcGCE	*Phanerochaete carnosa*	CaMV 35S	Hybrid Aspen Stems & Leaves	-	(Latha Gandla et al.)
PvPGIP1	Polygalacturonase (3.2.1.15)	Pga2	*Aspergillus niger*	SAG12	Arabidopsis Leaves	-	(Tomassetti et al., 2015)
	Pectate Lyase (4.2.2.2)	Pel2	*Pectobacterium carotovorum*	OlexA-46	Arabidopsis Leaves	-	(Tomassetti et al., 2015)

Signal Peptide / Terminator Sequence	Enzyme Activity	Enzyme Name (Traditional/ Family)	Enzyme Source Organism	Promoter	Plant Species and Tissue	% Total Soluble Protein	Ref.
Rice α-amylase 3A / 3D	Endo-1,4-β-glucanase (3.2.1.4)	E1/AcCel5A	*Acidothermus cellulolyticus*	CaMV 35S	Tobacco Leaves	0.4%	(Hwang et al., 2012)
				Pact2	Tobacco Leaves	<1%	(Hahn et al., 2014)
		E1D	Synthetic Construct (Dicot Optimized)	CaMV 35S	Sunflower Leaves	0.05%	(Jung et al., 2014)
	Endo-1,4-β-xylanase (3.2.1.8)	Xyl3	Synthetic Construct	CaMV 35S	Sunflower Leaves	0.07%	(Jung et al., 2014)
**Endoplasmic Reticulum**							
ALE / LKDEL	Feruloyl esterase (3.1.1.73)	AnFaeA	*Aspergillus niger*	Rice Actin 1	Tall Fescue Leaves	-	(Buanafina et al., 2010)
				Soybean Heat Shock	Tall Fescue Leaves	-	(Buanafina et al., 2010)
Arabidopsis 2S2 / DIKDEL	Endo-1,4-β-xylanase / Feruloyl esterase[a] (3.2.1.8 / 3.1.1.73)	XynZ/RtXyn10	*Ruminiclostridium thermocellum*	MMV	Tobacco Leaves	0.2 – 4.6%	(Chatterjee et al., 2010)
BAASS / KDEL	Endo-1,4-β-glucanase (3.2.1.4)	E1/AcCel5A	*Acidothermus cellulolyticus*	Glb1	Maize Seeds	17.9%	(Hood et al., 2007)
	Cellulose 1,4-β-cellobiosidase[R] (3.2.1.176)	CBHI/TrCel7A	*Trichoderma reesei*[c]	Glb1	Maize Seeds	16.3%	(Hood et al., 2007)
	Endo-1,4-β-xylanase (3.2.1.8)	XynA/BsXyn11	*Bacillus subtilis*	Wheat Glutenin 1DX5	Wheat Seeds	-	(Harholt et al., 2010)
	Feruloyl esterase (3.1.1.73)	AnFaeA	*Aspergillus niger*	Wheat Glutenin 1DX5	Wheat Seeds	-	(Harholt et al., 2010)
CALSP / HDEL	Endo-1,4-β-glucanase (3.2.1.4)	E2/TfCel6A	*Thermobifida fusca*	CaMV 35SS	Tobacco Leaves	-	(Jiang et al., 2011)
	Cellulose 1,4-β-cellobiosidase[NR] (3.2.1.91)	E3/TfCel6B	*Thermobifida fusca*	CaMV 35SS	Tobacco Leaves	-	(Jiang et al., 2011)

Signal Peptide / Terminator Sequence	Enzyme Activity	Enzyme Name (Traditional/ Family)	Enzyme Source Organism	Promoter	Plant Species and Tissue	% Total Soluble Protein	Ref.
**Endoplasmic Reticulum (cont.)**							
MMA / KDEL	Endo-1,4-β-glucanase (3.2.1.4)	SsCel12	*Sulfolobus solfataricus*	CaMV 35SS	Tobacco	-	(Klose et al., 2012)
		TrCel5A	*Trichoderma reesei*[c]	CaMV 35SS	Tobacco Leaves	-	(Klose et al., 2015)
Pr1b / KDEL	Polygalacturonase (3.2.1.15)	AnPgaI	*Aspergillus niger*	CaMV 35SS	Tobacco Leaves	3.0%	(Pereira et al., 2014)
	Feruloyl esterase (3.1.1.73)	AnFaeB	*Aspergillus niger*	tCUP4	Alfalfa Leaves	-	(Badhan et al., 2014)
SPER / KDEL	Endo-1,4-β-glucanase (3.2.1.4)	E1/AcCel5A	*Acidothermus cellulolyticus*	Mac	Tobacco Leaves	-	(Dai et al., 2005)
				RbcS1	Maize Leaves	0.2 – 2.0%	(Mei et al., 2009)
				RbcS1	Maize Leaves	0.2 – 2.0%	(Park et al., 2011)
ɣ-zein / KDEL or SEKDEL	Endo-1,4-β-glucanase (3.2.1.4)	EG/Cel5	Synthetic Construct	Maize PepC	Sugarcane Leaves	< 0.05%	(Harrison et al., 2011)
	Cellulose 1,4-β-cellobiosidase[R] (3.2.1.176)	CBHI/Cel7	Synthetic Construct	Maize Ubiquitin 1	Sugarcane Leaves	-	(Harrison et al., 2011)
				Maize PepC	Sugarcane Leaves	-	(Harrison et al., 2011) (Harrison et al., 2014b)
	Cellulose 1,4-β-cellobiosidase[NR] (3.2.1.91)	CBHII/Cel6	Synthetic Construct	Maize PepC	Sugarcane Leaves	-	(Harrison et al., 2011) (Harrison et al., 2014b)
	Endo-1,4-β-xylanase (3.2.1.8)	XynB/SoXyn11	*Streptomyces olivaceoviridis* A1	CaMV 35S	Tobacco Leaves	9%	(Llop-Tous et al., 2011)
**Chloroplast**							
-	Endo-1,4-β-glucanase (3.2.1.4)	E1/AcCel5A	*Acidothermus cellulolyticus*	Prrn	Tobacco Leaves, Stems & Roots	12%	(Ziegelhoffer et al., 2009)
		EGPh/PhCel5	*Pyrococcus horikoshii*	Prrn	Tobacco Leaves	25%	(Nakahira et al., 2013)

Signal Peptide / Terminator Sequence	Enzyme Activity	Enzyme Name (Traditional/ Family)	Enzyme Source Organism	Promoter	Plant Species and Tissue	% Total Soluble Protein	Ref.
**Chloroplast (cont.)**							
-	Endo-1,4-β-glucanase (3.2.1.4)	CelD/RtCel9A	*Ruminiclostridium thermocellum*	Prrn	Tobacco Leaves	-	(Verma et al., 2010)
		EG/TfCel6A	*Thermobifida fusca*	Prrn	Tobacco Leaves	2-3%	(Yu et al., 2007)
				Prrn	Tobacco Leaves	10.7%	(Gray et al., 2009)
		CelD/TfCel9A	*Thermobifida fusca*	Prrn	Tobacco Leaves	5~40% est.	(Petersen and Bock, 2011)
		EG3/TrCel12A	*Trichoderma reesei*[c]	Prrn	Tobacco Leaves	-	(Verma et al., 2010)
	Cellulose 1,4-β-cellobiosidase[NR] (3.2.1.91)	CelO/Cel5F	*Ruminiclostridium thermocellum*	Prrn	Tobacco Leaves	-	(Verma et al., 2010)
		CBHII/TfCel6B	*Thermobifida fusca*	Prrn	Tobacco Leaves	3-4%	(Yu et al., 2007)
				Prrn	Tobacco Leaves	~5% est.	(Petersen and Bock, 2011)
	1,4-β-glucosidase (3.2.1.21)	BglC/TfBgl1C	*Thermobifida fusca*	Prrn	Tobacco Leaves	8 – 12%	(Gray et al., 2011b)
				Prrn	Tobacco Leaves	-	(Petersen and Bock, 2011)
		Bgl1/TrCel3A	*Trichoderma reesei*	Prrn	Tobacco Leaves	-	(Verma et al., 2010)
				Prrn	Tobacco Leaves	-	(Jin et al., 2011)
	Xyloglucan-specific Endo-1,4-β-glucanase (3.2.1.151)	-/Xeg74	*Thermobifida fusca*	Prrn	Tobacco Leaves	-	(Petersen and Bock, 2011)
	Endo-1,4-β-xylanase (3.2.1.8)	AnXyn10A	*Aspergillus niger*	PpsbA	Tobacco Leaves	0.2%	(Kolotilin et al., 2013)
				PpsbA+T7g10	Tobacco Leaves	3.3%	(Kolotilin et al., 2013)
		AnXyn11A	*Aspergillus niger*	PpsbA	Tobacco Leaves	6.0%	(Kolotilin et al., 2013)
				PpsbA+T7g10	Tobacco Leaves	2.5%	(Kolotilin et al., 2013)
		XynA/BsXyn11	*Bacillus subtilis*	Prrn	Tobacco Leaves	-	(Pantaleoni et al., 2014)
		CcXynA	*Clostridium cellulovorans*	Prrn	Tobacco Leaves	-	(Kolotilin et al., 2013)
				PpsbA	Tobacco Leaves	0.5%	(Kolotilin et al., 2013)
		TmXyl10B	*Thermotoga maritima*	Prrn	Tobacco Leaves	13%	(Kim et al., 2011)
		XynI/TrXyn11	*Trichoderma reesei*[c]	Prrn	Tobacco Leaves	-	(Verma et al., 2010)

Signal Peptide / Terminator Sequence	Enzyme Activity	Enzyme Name (Traditional/ Family)	Enzyme Source Organism	Promoter	Plant Species and Tissue	% Total Soluble Protein	Ref.
**Chloroplast (cont.)**							
-	Endo-1,4-β-mannosidase (3.2.1.78)	TrMan5A	*Trichoderma reesei*[c]	PpsbA	Tobacco Leaves	-	(Agrawal et al., 2011)
	Pectate lyase (4.2.2.2)	PelA	*Fusarium solani*	Prrn	Tobacco Leaves	-	(Verma et al., 2010)
		PelB	*Fusarium solani*	Prrn	Tobacco Leaves	-	(Verma et al., 2010)
		PelD	*Fusarium solani*	Prrn	Tobacco Leaves	-	(Verma et al., 2010)
	Pectin Lyase (4.2.2.10)	PelA	Synthetic Construct based on *Streptomyces thermocarboxydus*	Prrn	Tobacco Leaves	-	(Espinoza-Sánchez et al., 2015)
	Acetyl xylan esterase (3.1.1.72)	Axe1	*Trichoderma reesei*	Prrn	Tobacco Leaves	-	(Verma et al., 2010)
	Lipase (3.1.1.3)	LipY	*Mycobacterium tuberculosis*	Prrn	Tobacco Leaves	-	(Verma et al., 2010)
	Cutinase (3.1.1.74)	CutA	*Fusarium solani*	Prrn	Tobacco Leaves	-	(Verma et al., 2010)
				PpsbA	Tobacco Leaves	-	(Verma et al., 2013)
	Swollenin	Swo1	*Trichoderma reesei*[c]	Prrn	Tobacco Leaves	-	(Verma et al., 2010)
				PpsbA	Tobacco Leaves	-	(Verma et al., 2013)
	Manganese peroxidase (1.11.1.13)	MnP2	Synthetic Construct based on *Phanerochaete chrysosporium*	Prrn	Tobacco Leaves	-	(Espinoza-Sánchez et al., 2015)
CAB	Endo-1,4-β-glucanase	TmCel5A	*Thermotoga maritima*	RbcSK-1A	Tobacco Leaves	3.8%	(Kim et al., 2010)
CTP	Cellulose 1,4-β-cellobiosidase[NR]	E3/TfCel6B	*Thermobifida fusca*	Pact2	Tobacco Leaves	25%	(Hahn et al., 2014)

Signal Peptide / Terminator Sequence	Enzyme Activity	Enzyme Name (Traditional/ Family)	Enzyme Source Organism	Promoter	Plant Species and Tissue	% Total Soluble Protein	Ref.
**Chloroplast (cont.)**							
Cyanophora paradoxa FNR	Endo-1,4-β-glucanase	Cel5	Synthetic Construct	Maize PepC	Sugarcane Leaves	< 0.05%	(Harrison et al., 2011; Harrison et al., 2014b)
RA	Endo-1,4-β-glucanase (3.2.1.4)	TmCel5A	*Thermotoga maritima*	RbcSK-1A	Tobacco Leaves	4.6%	(Kim et al., 2010)
				Alfalfa RbcSK-1A	Tobacco Leaves	1.7%	(Mahadevan et al., 2011)
				Alfalfa RbcSK-1A	Tobacco Leaves	1.8%	(Mahadevan et al., 2011)
	1,4-β-glucosidase (3.2.1.21)	BglB/TmBgl3	*Thermotoga maritima*	Alfalfa RbcSK-1A	Tobacco Leaves	5.8%	(Jung et al., 2010)
	Endo-1,4-β-xylanase (3.2.1.8)	XylII/TrXyn11A	*Trichoderma reesei*[c]	Alfalfa RbcSK-1A	Arabidopsis Leaves	3.0%	(Bae et al., 2006)
				CaMV 35S	Arabidopsis Leaves	3.2%	(Bae et al., 2008)
	Feruloyl esterase (3.1.1.73)	FaeB	*Aspergillus niger*	tCUP4	Alfalfa Leaves	-	(Badhan et al., 2014)
RbcS / RbcS-2A	Endo-1,4-β-glucanase (3.2.1.4)	E1/AcCel5A	*Acidothermus cellulolyticus*	Mac	Tobacco Leaves	-	(Dai et al., 2005)
				RbcS-3C	Tobacco Leaves	-	(Dai et al., 2005)
		TmCel5A	*Thermotoga maritima*	RbcSK-1A	Tobacco Leaves	5.2%	(Kim et al., 2010)
	1,4-β-glucosidase (3.2.1.21)	BglB/TmBgl3	*Thermotoga maritima*	Alfalfa RbcSK-1A	Tobacco Leaves	5.5%	(Jung et al., 2013)
				MRbcSK-1A	Tobacco Leaves	6.9%	(Jung et al., 2013)
				MRbcSK-1A + aps	Tobacco Leaves	9.3%	(Jung et al., 2013)
	Polygalacturonase (3.2.1.15)	PgaI	*Aspergillus niger*	CaMV 35SS	Tobacco Leaves	0%	(Pereira et al., 2014)

Signal Peptide / Terminator Sequence	Enzyme Activity	Enzyme Name (Traditional/ Family)	Enzyme Source Organism	Promoter	Plant Species and Tissue	% Total Soluble Protein	Ref.
**Chloroplast (cont.)**							
RbcS + RA	1,4-β-glucosidase / Endo-1,4-β-glucanase[b] (3.2.1.21 / 3.2.1.4)	BglB/TmBgl3 EG/TmCel5A	*Thermotoga maritima* *Thermotoga maritima*	CaMV 35S	Tobacco and Arabidopsis Leaves	-	(Lee et al., 2012)
	Endo-1,4-β-xylanase / Endo-1,4-β-glucanase[b] (3.2.1.8 / 3.2.1.4)	XynII/TrXyn11A EG/TmCel5A	*Trichoderma reesei*[c] *Thermotoga maritima*	CaMV 35S	Tobacco and Arabidopsis Leaves	-	(Lee et al., 2012)
	Cellulose 1,4-β-cellobiosidase[NR] / Endo-1,4-β-glucanase[b] (3.2.1.91 / 3.2.1.4)	E3/TfCel6B EG/TmCel5A	*Thermobifida fusca* *Thermotoga maritima*	CaMV 35S	Tobacco and Arabidopsis Leaves	-	(Lee et al., 2012)
**Vacuole**							
ALE	Endo-1,4-β-xylanase (3.2.1.8)	XynII/TrXyn11A	*Trichoderma reesei*[c]	Rice Actin 1	Tall Fescue Leaves	-	(Buanafina et al., 2012)
	Feruloyl esterase (3.1.1.73)	AnFaeA	*Aspergillus niger*	Modified Rice Actin	Tall Fescue Leaves	-	(Buanafina et al., 2015)
ALE / Frameshift KDEL	Feruloyl esterase (3.1.1.73)	AnFaeA	*Aspergillus niger*	Rice actin	Italian Ryegrass Leaves	-	(Buanafina et al., 2006)
				Rice actin	Tall Fescue Leaves	-	(Buanafina et al., 2008)
				Maize Ubiquitin	Tall Fescue Leaves	-	(Buanafina et al., 2008)
				Soybean Heat Shock	Tall Fescue Leaves	-	(Buanafina et al., 2008)
				LmSee1	Tall Fescue Leaves	-	(Buanafina et al., 2008)
Maize Proaleurain + VT	Endo-1,3(4)-β-glucanase (3.2.1.6)	Bgl7A	*Bispora* sp. MEY-1	Zm-leg1A	Maize Seeds	-	(Zhang et al., 2013)
	Endo-1,4-β-mannosidase (3.2.1.78)	Amn5A/Man5A	*Bispora* sp. MEY-1	Zm-leg1A	Maize Seeds	-	(Xu et al., 2013)

Signal Peptide / Terminator Sequence	Enzyme Activity	Enzyme Name (Traditional/ Family)	Enzyme Source Organism	Promoter	Plant Species and Tissue	% Total Soluble Protein	Ref.
**Vacuole (cont.)**							
Maize Proaleurain + VT	α-Galactosidase (3.2.1.22)	Aga-F57/Aga36	*Gibberella* sp. F57	Zm-leg1A	Maize Seeds	-	(Yang et al., 2015)
Pr1b / CTPP	Polygalacturonase (3.2.1.15)	AnPgaI	*Aspergillus niger*	CaMV 35SS	Tobacco Leaves	1.9%	(Pereira et al., 2014)
	Feruloyl esterase (3.1.1.73)	FaeB	*Aspergillus niger*	tCUP4	Alfalfa Leaves	-	(Badhan et al., 2014)
Sweet Potato Sporamin A	Endo-1,4-β-glucanase (3.2.1.4)	E1/AcCel5A	*Acidothermus cellulolyticus*	Mac	Tobacco Leaves	-	(Dai et al., 2005)
VT	Endo-1,4-β-glucanase	E1/AcCel5A	*Acidothermus cellulolyticus*	Glb1	Maize Seeds	16%	(Hood et al., 2012; Hood et al., 2007)
	Cellulose 1,4-β-cellobiosidase[R] (3.2.1.176)	CBHI/TrCel7A	*Trichoderma reesei*[c]	Glb1	Maize Seeds	0%	(Hood et al., 2012; Hood et al., 2007; Hood et al., 2014)
	1,4-β-glucosidase (3.2.1.21)	BglA/BfBgl3	*Butyrivibrio fibrisolvens*	CaMV 35S	Maize Leaves	0.9 – 3.1%	(Park et al., 2011)
γ-zein / DELKAEAK	Endo-1,4-β-glucanase (3.2.1.4)	Cel5	Synthetic Construct	Maize PepC	Sugarcane Leaves	< 0.05%	(Harrison et al., 2011; Harrison et al., 2014b)
	Cellulose 1,4-β-cellobiosidase[R] (3.2.1.176)	CBHI/Cel7	Synthetic Construct	Maize PepC	Sugarcane Leaves	-	(Harrison et al., 2011; Harrison et al., 2014b)
	Cellulose 1,4-β-cellobiosidase[R]			Maize PepC	Maize Leaves	-	(Harrison et al., 2014a)
	Cellulose 1,4-β-cellobiosidase[NR] (3.2.1.91)	CBHII/Cel6	Synthetic Construct	Maize PepC	Sugarcane Leaves	-	(Harrison et al., 2011; Harrison et al., 2014b)

Signal Peptide / Terminator Sequence	Enzyme Activity	Enzyme Name (Traditional/ Family)	Enzyme Source Organism	Promoter	Plant Species and Tissue	% Total Soluble Protein	Ref.
**Mitochondria**							
Cox IV	Endo-1,4-β-glucanase (3.2.1.4)	E1/AcCel5A	*Acidothermus cellulolyticus*	RbcS1	Maize Leaves	0.1 – 0.2%	(Mei et al., 2009)
**Golgi System**							
RST	Endo-1,4-β-xylanase (3.2.1.8)	XynII/TrXyn11A	*Trichoderma reesei*[c]	Rice Actin 1	Tall Fescue Leaves	-	(Buanafina et al., 2012)
RST / Frameshift KDEL	Feruloyl esterase (3.1.1.73)	AnFaeA	*Aspergillus niger*	Rice Actin 1	Tall Fescue Leaves	-	(Buanafina et al., 2010)
**Endosperm**							
Glub-4	Endo-1,4-β-xylanase (3.2.1.8)	BSX/Xyn10	*Bacillus* sp. NG-27	Glub-4	Maize Seeds	4.0%	(Gray et al., 2011a)
		XynB/CsXyn10B	*Clostridium stercorarium*	Glub-4	Maize Seeds	16.4%	(Gray et al., 2011a)
**Peroxisome**							
- / SKL	Endo-1,4-β-xylanase (3.2.1.8)	XylII/TrXyn11A	*Trichoderma reesei*[c]	Alfalfa RbcSK-1A	Arabidopsis Leaves	1.7%	(Bae et al., 2006)
**Multi-Compartment Targeting (Chloroplast & Peroxisome)**							
RA / SKL	Endo-1,4-β-xylanase (3.2.1.8)	XynII/TrXyn11A	*Trichoderma reesei*[c]	Alfalfa RbcSK-1A	Arabidopsis Leaves	4.8%	(Bae et al., 2006)

[R]Reducing end cellobiohydrolase
[NR]Non-reducing end cellobiohydrolase
[a]Multi-functional enzyme
[b]2A-mediated polyprotein
[c]*T. reesei* is the anamorph of *Hypocrea jecorina*

Table S2: Summary of heterologous production of cell wall degrading enzymes since 2005: Arranged by enzyme.

Abbreviations: ***alc*Amin35S**: alcohol inducible promoter based on CaMV 35S; **ALE**: barley aleurain vacuole targeting signal; **aps**: amplification promoting sequence; **BAASS**: Barley α-amylase signal sequence; **CAB:** Chlorophyll *a/b* binding protein; **CALSP:** tobacco calreticulin signal peptide; **CaMV35SS**: double CaMV 35S promoter; **Cox IV**: Yeast cytochrome c oxidase subunit; **CTP:** artificial dicot chloroplast targeting sequence; **CTPP**: C-terminal propeptide tobacco chitinase vacuolar sorting signal; **DELKAEAK**: vacuole sorting determinant; **FNR:** ferredoxin-$NADP^+$-oxidoreductase; **Frameshift KDEL**: frameshifted terminal peptide (ETTEG) that removes ER retention; **Glb1:** Maize globulin1; **Glb2**: globulin2; **Glub-4:** rice glutelin B-4 gene; **Gt1**: rice glutelin Gt1 promoter; **(SE/DI/L)KDEL/HDEL**: endoplasmic reticulum retention signal; **LmSee1**: Lolium multiflorium senescence enhanced gene promoter; **Mac**: hybrid of Ti plasmid mannopine synthetase promoter and cauliflower mosaic virus 35S promoter enhancer; **MMA:** leader peptide derived from murine monoclonal antibody mAb24; **MMV**: Mirabilis mosaic virus promoter; **MRbcSK-1A**: three alfalfa RbcS promoters (RbcSK-1A) without negative regulatory region; **OlexA-46**: β-estradiol inducible promoter; **Pact2:** Arabidopsis actin 2 promoter; **PepC**: phosphoenolpyruvate carboxylase; **PpsbA:** PSII protein D1 promoter; **PPI**: Potato protease inhibitor II; **Prrn**: Tobacco 16S ribosomal ERNA promoter; **PR-S/PR1a/PR1b**: pathogenesis-related proteins; **PvPGIP1:** *P. vulgaris* polygalacturonase-inhibiting protein ; **RA**: Rubisco activase; **RbcS**: Rubisco small subunit; **Rice SGR**: Rice Stay Green gene; **RST**: rat sialyl transferase Golgi targeting motif; **Rubi3**: rice ubiquitin promoter; **SAG12:** Arabidopsis senescence inducible promoter; **SKL**: peroxisome targeting C-terminal sequence; **SPER**: endoplasmic reticulum targeting signal peptide; **T7g10**: bacteriophage T7 gene 10 N-terminal enhancer; **VT:** vacuole targeting signal peptide; **Zm-leg1A**: maize legumin promoter

Enzyme Source Organism	Enzyme Name (Traditional/ Family)	Targeting Location	Signal Peptide / Terminator Sequence	Promoter	Plant Species and Tissue	% Total Soluble Protein	Ref.
**Endo-1,4-β-glucanase (3.2.1.4)**							
Acidothermus cellulolyticus	E1/AcCel5A	Cytosol	-	Mac	Tobacco Leaves	-	(Dai et al., 2005)
				CaMV 35S	Duckweed Plants	0.24%	(Sun et al., 2007)
				Gt1	Rice Seeds	-	(Zhang et al., 2012)
		Apoplast	BAASS	Glb1	Maize Seeds	ND	(Hood et al., 2007)
				Glb1	Maize Seeds	7.1%	(Egelkrout et al., 2013)
				Glb2 + Glb1 + pr26	Maize Seeds	18.8%	(Egelkrout et al., 2013)
			Tobacco PR-S	Mac	Tobacco Leaves	-	(Dai et al., 2005)

Enzyme Source Organism	Enzyme Name (Traditional/ Family)	Targeting Location	Signal Peptide / Terminator Sequence	Promoter	Plant Species and Tissue	% Total Soluble Protein	Ref.
**Endo-1,4-β-glucanase (3.2.1.4) cont…**							
Acidothermus cellulolyticus	E1/AcCel5A	Apoplast	Tobacco Pr1a	CaMV 35S	Maize Leaves and Roots	2.1%	(Biswas et al., 2006)
				CaMV 35S	Maize Leaves	1.2%	(Ransom et al., 2007)
				CaMV 35S	Rice Leaves	4.9%	(Oraby et al., 2007)
				Mac	Rice Leaves	6.1%	(Chou et al., 2011)
			Rice α-amylase	CaMV 35S	Tobacco Leaves	0.4%	(Hwang et al., 2012)
			Rice α-amylase 3A	Pact2	Tobacco Leaves	<1%	(Hahn et al., 2014)
		ER	BAASS / KDEL	Glb1	Maize Seeds	17.9%	(Hood et al., 2007)
			SPER / KDEL	Mac	Tobacco Leaves	-	(Dai et al., 2005)
			SPER / ER Retention Signal	RbcS1	Maize Leaves	0.2 – 2.0%	(Mei et al., 2009)
				RbcS1	Maize Leaves	0.2 – 2.0%	(Park et al., 2011)
		Chloroplast	-	Prrn	Tobacco Leaves, Stems & Roots	12%	(Ziegelhoffer et al., 2009)
			RbcS-2A	Mac	Tobacco Leaves	-	(Dai et al., 2005)
				RbcS-3C	Tobacco Leaves	-	(Dai et al., 2005)
		Mitochondria	SPM	RbcS1	Maize Leaves	0.1 – 0.2%	(Mei et al., 2009)
		Vacuole	Sweet Potato Sporamin A	Mac	Tobacco Leaves	-	(Dai et al., 2005)
			VT	Glb1	Maize Seeds	16%	(Hood et al., 2012; Hood et al., 2007)

Enzyme Source Organism	Enzyme Name (Traditional/ Family)	Targeting Location	Signal Peptide / Terminator Sequence	Promoter	Plant Species and Tissue	% Total Soluble Protein	Ref.
**Endo-1,4-β-glucanase (3.2.1.4) cont…**							
Oryza sativa	ENG1/-	Cytosol	-	Maize Ubiquitin	Rice Leaves	-	(Nigorikawa et al., 2012)
Pyrococcus horikoshii	EGPh/PhCel5	Chloroplast	-	Prrn	Tobacco Leaves	25%	(Nakahira et al., 2013)
Ruminiclostridium thermocellum	CelD/RtCel9A	Chloroplast	-	Prrn	Tobacco Leaves	-	(Verma et al., 2010)
Sulfolobus solfataricus	SsCel12	Apoplast	MMA	CaMV 35SS	Tobacco	-	(Klose et al., 2012)
		ER	MMA / KDEL	CaMV 35SS	Tobacco	-	(Klose et al., 2012)
Thermobifida fusca	E2/TfCel6A	Cytosol	-	CaMV 35SS	Tobacco Leaves	-	(Jiang et al., 2011)
		Apoplast	CALSP	CaMV 35SS	Tobacco Leaves	-	(Jiang et al., 2011)
		ER	CALSP / HDEL	CaMV 35SS	Tobacco Leaves	-	(Jiang et al., 2011)
		Chloroplast	-	Prrn	Tobacco Leaves	2-3%	(Yu et al., 2007)
						10.7%	(Gray et al., 2009)
	CelD/TfCel9A	Chloroplast	-	Prrn	Tobacco Leaves	5~40% est.	(Petersen and Bock, 2011)
Thermotoga maritima	EG/TmCel5A	Cytosol	-	Alfalfa RbcSK-1A	Tobacco Leaves	0%	(Mahadevan et al., 2011)
				Pact2	Tobacco Leaves	12.5%	(Hahn et al., 2014)
		Apoplast	Tobacco PR1a	Alfalfa RbcSK-1A	Tobacco Leaves	1.2%	(Mahadevan et al., 2011)
		Chloroplast	CAB	RbcSK-1A	Tobacco Leaves	3.8%	(Kim et al., 2010)
			RbcS	RbcSK-1A	Tobacco Leaves	5.2%	(Kim et al., 2010)
			RA	RbcSK-1A	Tobacco Leaves	4.6%	(Kim et al., 2010)
				Alfalfa RbcSK-1A	Tobacco Leaves	1.7%	(Mahadevan et al., 2011)

Enzyme Source Organism	Enzyme Name (Traditional/ Family)	Targeting Location	Signal Peptide / Terminator Sequence	Promoter	Plant Species and Tissue	% Total Soluble Protein	Ref.
**Endo-1,4-β-glucanase (3.2.1.4) cont…**							
Trichoderma reesei[c]	EG/TrCel5A	Apoplast	MMA	*alc*Amin35S	Tobacco Leaves	-	(Klose et al., 2013)
				CaMV 35SS	Tobacco Leaves	-	(Klose et al., 2013)
				CaMV 35SS	Tobacco Leaves	-	(Klose et al., 2015)
		ER	MMA / KDEL	CaMV 35SS	Tobacco Leaves	-	(Klose et al., 2015)
	EG3/TrCel12A	Chloroplast	-	Prrn	Tobacco Leaves	-	(Verma et al., 2010)
Synthetic Construct (Dicot Optimized)	E1D	Apoplast	Rice α-amylase 3D	CaMV 35S	Sunflower Leaves	0.05%	(Jung et al., 2014)
Synthetic Construct	EG/Cel5	ER	ɣ-zein / SEKDEL	Maize PepC	Sugarcane Leaves	< 0.05%	(Harrison et al., 2011)
		Chloroplast	*Cyanophora paradoxa FNR*	Maize PepC	Sugarcane Leaves	< 0.05%	(Harrison et al., 2011) (Harrison et al., 2014b)
		Vacuole	ɣ-zein / DELKAEAK	Maize PepC	Sugarcane Leaves	< 0.05%	(Harrison et al., 2011) (Harrison et al., 2014b)
**Cellulose 1,4-β-cellobiosidase[R] (3.2.1.176)**							
Humicola grisea	CBHI/HgCel7A	Apoplast	BAASS	Pact2	Tobacco Leaves	10%	(Hahn et al., 2014)
Trichoderma reesei[c]	CBHI/TrCel7A	Apoplast	BAASS	Glb1	Maize Seeds	17.8%	(Hood et al., 2007)
				Glb1	Maize Seeds	3.2%	(Egelkrout et al., 2013)
				Glb1 + pr36 + Glb2	Maize Seeds	4.1%	(Egelkrout et al., 2013)
				Pact2	Tobacco Leaves	12.5%	(Hahn et al., 2014)
			Tobacco Pr1a	CaMV 35S	Maize and Tobacco Leaves	-	(Park et al., 2011)
		ER	BAASS / KDEL	Glb1	Maize Seeds	16.3%	(Hood et al., 2007)
		Vacuole	VT	Glb1	Maize Seeds	0%	(Hood et al., 2012; Hood et al., 2007; Hood et al., 2014)

Enzyme Source Organism	Enzyme Name (Traditional/ Family)	Targeting Location	Signal Peptide / Terminator Sequence	Promoter	Plant Species and Tissue	% Total Soluble Protein	Ref.
**Cellulose 1,4-β-cellobiosidase[R] (3.2.1.176) cont…**							
Synthetic Construct	CBHI/Cel7	ER	ɣ-zein / SEKDEL	Maize Ubi1	Sugarcane Leaves	-	(Harrison et al., 2011)
				Maize PepC	Sugarcane Leaves	-	(Harrison et al., 2011) (Harrison et al., 2014b)
		Vacuole	ɣ-zein / DELKAEAK	Maize PepC	Sugarcane Leaves	-	(Harrison et al., 2011; Harrison et al., 2014b)
					Maize Leaves	-	(Harrison et al., 2014a)
**Cellulose 1,4-β-cellobiosidase[NR] (3.2.1.91)**							
Oryza sativa	EXG1/-	Cytosol	-	Maize Ubiquitin	Rice Leaves	-	(Nigorikawa et al., 2012)
				Rice SGR	Rice Straw	-	(Furukawa et al., 2014)
Ruminiclostridium thermocellum	CelO/RtCel5F	Chloroplast	-	Prrn	Tobacco Leaves	-	(Verma et al., 2010)
Thermobifida fusca	E3/TfCel6B	Cytosol	-	CaMV35SS	Tobacco Leaves	-	(Jiang et al., 2011)
		Apoplast	CALSP	CaMV35SS	Tobacco Leaves	-	(Jiang et al., 2011)
		ER	CALSP / HDEL	CaMV35SS	Tobacco Leaves	-	(Jiang et al., 2011)
		Chloroplast	-	Prrn	Tobacco Leaves	3-4%	(Yu et al., 2007)
						~5% est.	(Petersen and Bock, 2011)
			CTP	Pact2	Tobacco Leaves	25%	(Hahn et al., 2014)
Trichoderma reesei[c]	CBHII/TrCel6A	Apoplast	BAASS	Rice Glutelin	Maize Seeds	30%	(Devaiah et al., 2013)
Synthetic Construct	CBHII/Cel6	ER	ɣ-zein / SEKDEL	Maize PepC	Sugarcane Leaves	-	(Harrison et al., 2011) (Harrison et al., 2014b)
		Vacuole	ɣ-zein / DELKAEAK	Maize PepC	Sugarcane Leaves	-	(Harrison et al., 2011; Harrison et al., 2014b)

Enzyme Source Organism	Enzyme Name (Traditional/ Family)	Targeting Location	Signal Peptide / Terminator Sequence	Promoter	Plant Species and Tissue	% Total Soluble Protein	Ref.
**1,4-β-glucosidase (3.2.1.21)**							
Butyrivibrio fibrisolvens	BglA/BfBgl3	Vacuole	VT	CaMV 35S	Maize Leaves	0.9 – 3.1%	(Park et al., 2011)
Humicola grisea	HgBgl4	Cytosol	-	Pact2	Tobacco Leaves	50%	(Hahn et al., 2014)
Oryza sativa	BEG1/-	Cytosol	-	Rice actin	Rice Leaves	-	(Nigorikawa et al., 2012)
Thermobifida fusca	BglC/TfBgl1C	Chloroplast	-	Prrn	Tobacco Leaves	8 – 12%	(Gray et al., 2011b)
						-	(Petersen and Bock, 2011)
Thermotoga maritima	BglB/TmBgl3	Cytosol	-	Alfalfa RbcSK-1A	Tobacco Leaves	4.5%	(Jung et al., 2010; Jung et al., 2013)
				MRbcSK-1A + aps	Tobacco Leaves	8.3%	(Jung et al., 2013)
		Chloroplast	RA	Alfalfa RbcSK-1A	Tobacco Leaves	5.8%	(Jung et al., 2010)
			RbcS	Alfalfa RbcSK-1A	Tobacco Leaves	5.5%	(Jung et al., 2013)
				MRbcSK-1A	Tobacco Leaves	6.9%	(Jung et al., 2013)
				MRbcSK-1A + aps	Tobacco Leaves	9.3%	(Jung et al., 2013)
Trichoderma reesei	Bgl1/TrCel3A	Chloroplast	-	Prrn	Tobacco Leaves	-	(Verma et al., 2010)
			-	Prrn	Tobacco Leaves	-	(Jin et al., 2011)
**Endo-1,3(4)-β-glucanase (3.2.1.6)**							
Bispora sp. MEY-1	Bgl7A	Vacuole	Maize Proaleurain + VT	Zm-leg1A	Maize Seeds	-	(Zhang et al., 2013)
**Xyloglucan-specific Endo-1,4-β-glucanase (3.2.1.151)**							
Thermobifida fusca	-/Xeg74	Chloroplast	-	Prrn	Tobacco Leaves	-	(Petersen and Bock, 2011)

Enzyme Source Organism	Enzyme Name (Traditional/ Family)	Targeting Location	Signal Peptide / Terminator Sequence	Promoter	Plant Species and Tissue	% Total Soluble Protein	Ref.
**Endo-1,4-β-xylanase (3.2.1.8)**							
Aspergillus niger	AnXyn10A	Chloroplast	-	PpsbA	Tobacco Leaves	0.2%	(Kolotilin et al., 2013)
				PpsbA+T7g10	Tobacco Leaves	3.3%	(Kolotilin et al., 2013)
	AnXyn11A	Chloroplast	-	PpsbA	Tobacco Leaves	6.0%	(Kolotilin et al., 2013)
				PpsbA+T7g10	Tobacco Leaves	2.5%	(Kolotilin et al., 2013)
Bacillus sp. NG-27	BSX/Xyn10	Apoplast	BAASS	Rubi3	Maize Stover	0.1%	(Gray et al., 2011a)
		Endosperm	Glub-4	Glub-4	Maize Seeds	4.0%	(Gray et al., 2011a)
Bacillus subtilis	XynA/BsXyn11	Apoplast	BAASS	Wheat Glutenin 1DX5	Wheat Seeds	-	(Harholt et al., 2010)
		ER	BAASS / KDEL	Wheat Glutenin 1DX5	Wheat Seeds	-	(Harholt et al., 2010)
		Chloroplast	-	Prrn	Tobacco Leaves	-	(Pantaleoni et al., 2014)
Clostridium cellulovorans	XynA	Chloroplast	-	Prrn	Tobacco Leaves	-	(Kolotilin et al., 2013)
				PpsbA	Tobacco Leaves	0.5%	(Kolotilin et al., 2013)
Clostridium stercorarium	XynB/CsXyn10B	Cytosol	-	CaMV 35S	Tobacco Leaves	0.1%	(Kimura et al., 2010)
				Rice actin act1	Rice Leaves	0.1 – 0.2%	(Kimura et al., 2010)
		Apoplast	BAASS	Rubi3	Maize Stover	0.1%	(Gray et al., 2011a)
		Endosperm	Glub-4	Glub-4	Maize Seeds	16.4%	(Gray et al., 2011a)
Dictyoglomus thermophilum	XynA/DtXyn10	Apoplast	BAASS	CaMV 35S	Arabidopsis Leaves and Stems	14%	(Borkhardt et al., 2010)
	XynB/DtXyn11	Apoplast	BAASS	CaMV 35S	Arabidopsis Leaves and Stems	3%	(Borkhardt et al., 2010)
				Rice Ubi3	Maize Seeds	-	(Shen et al., 2012)

Enzyme Source Organism	Enzyme Name (Traditional/ Family)	Targeting Location	Signal Peptide / Terminator Sequence	Promoter	Plant Species and Tissue	% Total Soluble Protein	Ref.
**Endo-1,4-β-xylanase (3.2.1.8) cont…**							
Streptomyces olivaceoviridis A1	XynB/SoXyn11	Cytosol	-	CaMV 35SS	Potato Leaves and Tubers	5%	(Yang et al., 2007)
		Apoplast	PPI	CaMV 35SS	Potato Leaves and Tubers	-	(Yang et al., 2007)
		ER (Protein Bodies)	γ-zein / SEKDEL	CaMV 35S	Tobacco Leaves	9%	(Llop-Tous et al., 2011)
Thermotoga maritima	TmXyl10B	Chloroplast	-	Prrn	Tobacco Leaves	13%	(Kim et al., 2011)
Trichoderma reesei[c]	XynI/TrXyn11	Chloroplast	-	Prrn	Tobacco Leaves	-	(Verma et al., 2010)
	XylII/TrXyn11A	Cytosol	-	Alfalfa RbcSK-1A	Arabidopsis Leaves	1.2%	(Bae et al., 2006)
				CaMV 35S	Arabidopsis Leaves	1.4%	(Bae et al., 2008)
		Apoplast	PPI	Rice Actin 1	Tall Fescue Leaves	-	(Buanafina et al., 2012)
				LmSee1	Tall Fescue Leaves	-	(Buanafina et al., 2015; Buanafina et al., 2012)
		Chloroplast	RA	Alfalfa RbcSK-1A	Arabidopsis Leaves	3.0%	(Bae et al., 2006)
				CaMV 35S	Arabidopsis Leaves	3.2%	(Bae et al., 2008)
		Vacuole	ALE	Rice Actin 1	Tall Fescue Leaves	-	(Buanafina et al., 2012)
		Golgi System	RST	Rice Actin 1	Tall Fescue Leaves	-	(Buanafina et al., 2012)
		Peroxisome	- / SKL	Alfalfa RbcSK-1A	Arabidopsis Leaves	1.7%	(Bae et al., 2006)
		Chloroplast & Peroxisome	RA / SKL	Alfalfa RbcSK-1A	Arabidopsis Leaves	4.8%	(Bae et al., 2006)

Enzyme Source Organism	Enzyme Name (Traditional/ Family)	Targeting Location	Signal Peptide / Terminator Sequence	Promoter	Plant Species and Tissue	% Total Soluble Protein	Ref.
**Endo-1,4-β-xylanase (3.2.1.8) cont…**							
Synthetic Construct	ATX/Xyn11	Cytosol	-	CaMV 35S	Rice Leaves	-	(Weng et al., 2013)
	Xyl3	Apoplast	Rice α-amylase 3D	CaMV 35S	Sunflower Leaves	0.07%	(Jung et al., 2014)
**Endo-1,4-β-mannosidase (3.2.1.78)**							
Bacillus sp. JAMB-602	Amn5A/Man5A	Apoplast	Chitinase 1	CaMV 35S	Tobacco Leaves	-	(Hoshikawa et al., 2012)
Bispora sp. MEY-1	Man5A	Vacuole	Maize Proaleurain + VT	Zm-leg1A	Maize Seeds	-	(Xu et al., 2013)
Trichoderma reesei[c]	TrMan5A	Chloroplast	-	PpsbA	Tobacco Leaves	-	(Agrawal et al., 2011)
**α-Galactosidase (3.2.1.22)**							
Gibberella sp. F57	Aga-F57/Aga36	Vacuole	Maize Proaleurain + VT	Zm-leg1A	Maize Seeds	-	(Yang et al., 2015)
**Polygalacturonase (3.2.1.15)**							
Aspergillus niger	PgaI	Cytosol	-	CaMV 35SS	Tobacco Leaves	0%	(Pereira et al., 2014)
		Apoplast	Pr1b	CaMV 35SS	Tobacco Leaves	2.5%	(Pereira et al., 2014)
		ER	Pr1b / KDEL	CaMV 35SS	Tobacco Leaves	3.0%	(Pereira et al., 2014)
		Chloroplast	RbsC	CaMV 35SS	Tobacco Leaves	0%	(Pereira et al., 2014)
		Vacuole	Pr1b / CTPP	CaMV 35SS	Tobacco Leaves	1.9%	(Pereira et al., 2014)
	Pga2	Apoplast	SAG12	PvPGIP1	Arabidopsis Leaves	-	(Tomassetti et al., 2015)
**Pectate Lyase (4.2.2.2)**							
Fusarium solani	PelA	Chloroplast	-	Prrn	Tobacco Leaves	-	(Verma et al., 2010)
	PelB	Chloroplast	-	Prrn	Tobacco Leaves	-	(Verma et al., 2010)
	PelD	Chloroplast	-	Prrn	Tobacco Leaves	-	(Verma et al., 2010)
Pectobacterium carotovorum	Pel2	Apoplast	OlexA-46	PvPGIP1	Arabidopsis Leaves	-	(Tomassetti et al., 2015)

Enzyme Source Organism	Enzyme Name (Traditional/ Family)	Targeting Location	Signal Peptide / Terminator Sequence	Promoter	Plant Species and Tissue	% Total Soluble Protein	Ref.
**Pectin Lyase (4.2.2.10)**							
Synthetic Construct	PelA	Chloroplast	-	Prrn	Tobacco Leaves	-	(Espinoza-Sánchez et al., 2015)
**Acetylxylan esterase (3.1.1.72)**							
Aspergillus nidulans	AnAXE	Apoplast	Arabidopsis β - expansin	CaMV 35S	Arabidopsis Leaves & Stems	-	(Pogorelko et al., 2011; Pogorelko et al., 2013)
			Maize expansin B	Maize Ubiquitin	Brachypodium Leaves	-	(Pogorelko et al., 2013)
Trichoderma reesei	Axe1	Chloroplast	-	Prrn	Tobacco Leaves	-	(Verma et al., 2010)
**Feruloyl esterase (3.1.1.73)**							
Aspergillus nidulans	AnFAE	Apoplast	Arabidopsis β - expansin	CaMV 35S	Arabidopsis Leaves & Stems	-	(Pogorelko et al., 2011)
Aspergillus niger	FaeA	Apoplast	PPI	Rice Actin 1	Tall Fescue Leaves	-	(Buanafina et al., 2015; Buanafina et al., 2010)
			ALE	Rice Actin 1	Tall Fescue Leaves	-	(Buanafina et al., 2010)
				Soybean Heat Shock	Tall Fescue Leaves	-	(Buanafina et al., 2010)
			ALE / Frameshift KDEL	LmSee1	Tall Fescue Leaves	-	(Buanafina et al., 2010)
			BAASS	Wheat Glutenin 1DX5	Wheat Seeds	-	(Harholt et al., 2010)
		ER	BAASS / KDEL	Wheat Glutenin 1DX5	Wheat Seeds	-	(Harholt et al., 2010)
			ALE / LKDEL	Rice Actin 1	Tall Fescue Leaves	-	(Buanafina et al., 2010)
				Soybean Heat Shock	Tall Fescue Leaves	-	(Buanafina et al., 2010)

Enzyme Source Organism	Enzyme Name (Traditional/ Family)	Targeting Location	Signal Peptide / Terminator Sequence	Promoter	Plant Species and Tissue	% Total Soluble Protein	Ref.
**Feruloyl esterase (3.1.1.73) cont…**							
Aspergillus niger	FaeA	Vacuole	ALE	Modified Rice Actin	Tall Fescue Leaves	-	(Buanafina et al., 2015)
			ALE / Frameshift KDEL	Rice actin	Italian Ryegrass Leaves	-	(Buanafina et al., 2006)
				Rice actin	Tall Fescue Leaves	-	(Buanafina et al., 2008)
				Maize Ubiquitin	Tall Fescue Leaves	-	(Buanafina et al., 2008)
				Soybean Heat Shock	Tall Fescue Leaves	-	(Buanafina et al., 2008)
				LmSee1	Tall Fescue Leaves	-	(Buanafina et al., 2008)
			BAVT / Frameshift KDEL	CaMV 35S	Tall Fescue Leaves	-	(Buanafina et al., 2008)
		Golgi System	RST / Frameshift KDEL	Rice Actin 1	Tall Fescue Leaves	-	(Buanafina et al., 2010)
	FaeB	Apoplast	PR1b	tCUP4	Alfalfa Leaves	-	(Badhan et al., 2014)
		ER	PR1b / KDEL	tCUP4	Alfalfa Leaves	-	(Badhan et al., 2014)
		Chloroplast	RA	tCUP4	Alfalfa Leaves	-	(Badhan et al., 2014)
		Vacuole	PR1b / CTPP	tCUP4	Alfalfa Leaves	-	(Badhan et al., 2014)
**Rhamnogalacturonan acetyl esterase (3.1.1.86)**							
Aspergillus nidulans	AnRAE	Apoplast	Arabidopsis β -expansin	CaMV 35S	Arabidopsis Leaves	-	(Pogorelko et al., 2013)
			Maize expansin B	Maize Ubiquitin	Brachypodium Leaves	-	(Pogorelko et al., 2013)

Enzyme Source Organism	Enzyme Name (Traditional/ Family)	Targeting Location	Signal Peptide / Terminator Sequence	Promoter	Plant Species and Tissue	% Total Soluble Protein	Ref.
**4-O-methyl-glucuronoyl methylesterase (3.1.1.-)**							
Phanerochaete carnosa	PcGCE	Cytosol	-	CaMV 35S	Arabidopsis Stems and Leaves	-	(Tsai et al., 2012)
		Apoplast	PttCel9B3	CaMV 35S	Hybrid Aspen Stems & Leaves	-	(Latha Gandla et al.)
**Lipase (3.1.1.3)**							
Mycobacterium tuberculosis	LipY	Chloroplast	-	Prrn	Tobacco Leaves	-	(Verma et al., 2010)
**Cutinase (3.1.1.74)**							
Fusarium solani	CutA	Chloroplast	-	Prrn	Tobacco Leaves	-	(Verma et al., 2010)
			-	PpsbA	Tobacco Leaves	-	(Verma et al., 2013)
**Laccase (1.10.3.2) + CBM**							
Trametes versicolor	Cvl3 + CBM	Cytosol	-	Ubiquitin	Rice Culm	-	(Furukawa et al., 2013)
**Manganese Peroxidase (1.11.1.13)**							
Phanerochaete chrysosporium	MnP2	Apoplast	BAASS	Glb1	Maize Seeds	6-14%	(Clough et al., 2006)
Synthetic Construct	MnP2	Chloroplast	-	Prrn	Tobacco Leaves	-	(Espinoza-Sánchez et al., 2015)
**Swollenin**							
Trichoderma reesei[c]	Swo1	Chloroplast	-	Prrn	Tobacco Leaves	-	(Verma et al., 2010)
		Chloroplast	-	PpsbA	Tobacco Leaves	-	(Verma et al., 2013)
**Endo-1,4-β-xylanase (3.2.1.8) + Feruloyl esterase (3.1.1.73)**[a]							
Ruminiclostridium thermocellum	XynZ/RtXyn10	Cytosol	-	MMV	Tobacco Leaves	1.5 – 3.1%	(Chatterjee et al., 2010)
		Apoplast	Arabidopsis 2S2	MMV	Tobacco Leaves	2.2 – 4.1%	(Chatterjee et al., 2010)
		ER	Arabidopsis 2S2 / DIKDEL	MMV	Tobacco Leaves	0.2 – 4.6%	(Chatterjee et al., 2010)

Enzyme Source Organism	Enzyme Name (Traditional/ Family)	Targeting Location	Signal Peptide / Terminator Sequence	Promoter	Plant Species and Tissue	% Total Soluble Protein	Ref.
***Chimeric:* Endo-1,4-β-xylanase (3.2.1.8) + Feruloyl esterase (3.1.1.73) + α-arabinofuranosidase (3.2.1.55)[a]**							
Ruminiclostridium thermocellum (Xyn/Fae); Geobacillus stearothermophilus (Abf)	XynZ/RtXyn10; AbfA/GsAbf51	Cytosol	-	MMV	Tobacco Leaves	0.6 – 2.0%	(Fan and Yuan, 2010)
**Endo-1,4-β-glucanase (3.2.1.4) + 1,4-β-glucosidase (3.2.1.21) [b]**							
Thermotoga maritima	BglB/TmBgl3 EG/TmCel5A	Chloroplast	RbcS RA	CaMV 35S	Tobacco and Arabidopsis Leaves	-	(Lee et al., 2012)
**Endo-1,4-β-glucanase (3.2.1.4) + Endo-1,4-β-xylanase (3.2.1.8) [b]**							
Trichoderma reesei[c] *Thermotoga maritima*	XynII/TrXyn11A EG/TmCel5A	Chloroplast	RbcS RA	CaMV 35S	Tobacco and Arabidopsis Leaves	-	(Lee et al., 2012)
**Endo-1,4-β-glucanase (3.2.1.4) + Cellulose 1,4-β-cellobiosidase[NR] (3.2.1.91) [b]**							
Thermobifida fusca Thermotoga maritima	E3/TfCel6B EG/TmCel5A	Chloroplast	RbcS RA	CaMV 35S	Tobacco and Arabidopsis Leaves	-	(Lee et al., 2012)

[R]Reducing end cellobiohydrolase
[NR]Non-reducing end cellobiohydrolase
[a]Multi-functional enzyme
[b]2A-mediated polyprotein
[c]*T. reesei* is the anamorph of *Hypocrea jecorina*

References

Abbott, D.W., Thomas, D., Pluvinage, B. and Boraston, A.B. (2013) An ancestral member of the polysaccharide lyase Family 2 displays endolytic activity and magnesium dependence. *Appl. Biochem. Biotechnol.* **171**, 1911-1923.

Agger, J.W., Isaksen, T., Várnai, A., Vidal-Melgosa, S., Willats, W.G.T., Ludwig, R., Horn, S.J., Eijsink, V.G.H. and Westereng, B. (2014) Discovery of LPMO activity on hemicelluloses shows the importance of oxidative processes in plant cell wall degradation. *Proc. Natl. Acad. Sci. U.S.A.* **111**, 6287-6292.

Agrawal, P., Verma, D. and Daniell, H. (2011) Expression of *Trichoderma reesei* β-mannanase in tobacco chloroplasts and its utilization in lignocellulosic woody biomass hydrolysis. *PLoS One* **6**, e29302.

Apgar, J., Ross, M., Zuo, X., Dohle, S., Sturtevant, D., Shen, B.Z., de la Vega, H., Lessard, P., Lazar, G. and Raab, R.M. (2012) A predictive model of intein insertion site for use in the engineering of molecular switches. *PLoS One* **7**, e37355.

Badhan, A., Jin, L., Wang, Y., Han, S., Kowalczys, K., Brown, D., Ayala, C., Latoszek-Green, M., Miki, B., Tsang, A. and McAllister, T. (2014) Expression of a fungal ferulic acid esterase in alfalfa modifies cell wall digestibility. *Biotechnol. Biofuels* **7**, 39.

Bae, H., Lee, D.-S. and Hwang, I. (2006) Dual targeting of xylanase to chloroplasts and peroxisomes as a means to increase protein accumulation in plant cells. *J. Exp. Bot.* **57**, 161-169.

Bae, H.-J., Kim, H.J. and Kim, Y.S. (2008) Production of a recombinant xylanase in plants and its potential for pulp biobleaching applications. *Bioresour. Technol.* **99**, 3513-3519.

Banerjee, G., Car, S., Scott-Craig, J., Borrusch, M. and Walton, J. (2010) Rapid optimization of enzyme mixtures for deconstruction of diverse pretreatment/biomass feedstock combinations. *Biotechnol. Biofuels* **3**, 22.

Biely, P. (2012) Microbial carbohydrate esterases deacetylating plant polysaccharides. *Biotechnol. Adv.* **30**, 1575-1588.

Biswas, G.C.G., Ransom, C. and Sticklen, M. (2006) Expression of biologically active *Acidothermus cellulolyticus* endoglucanase in transgenic maize plants. *Plant Sci.* **171**, 617-623.

Black, G.W., Rixon, J.E., Clarke, J.H., Hazlewood, G.P., Theodorou, M.K., Morris, P. and Gilbert, H.J. (1996) Evidence that linker sequences and cellulose-binding domains enhance the activity of hemicellulases against complex substrates. *Biochem. J.* **319**, 515-520.

Bonnin, E., Garnier, C. and Ralet, M.-C. (2014) Pectin-modifying enzymes and pectin-derived materials: applications and impacts. *Appl. Microbiol. Biotechnol.* **98**, 519-532.

Borkhardt, B., Harholt, J., Ulvskov, P., Ahring, B.K., Jørgensen, B. and Brinch-Pedersen, H. (2010) Autohydrolysis of plant xylans by apoplastic expression of thermophilic bacterial endo-xylanases. *Plant Biotechnol. J.* **8**, 363-374.

Buanafina, M.M.d.O., Dalton, S., Langdon, T., Timms-Taravella, E., Shearer, E.A. and Morris, P. (2015) Functional co-expression of a fungal ferulic acid esterase and a β-1,4 endoxylanase in *Festuca arundinacea* (tall fescue) modifies post-harvest cell wall deconstruction. *Planta* **242**, 97-111.

Buanafina, M.M.d.O., Langdon, T., Dalton, S. and Morris, P. (2012) Expression of a *Trichoderma reesei* β-1,4 endo-xylanase in tall fescue modifies cell wall structure and digestibility and elicits pathogen defence responses. *Planta* **236**, 1757-1774.

Buanafina, M.M.d.O., Langdon, T., Hauck, B., Dalton, S. and Morris, P. (2008) Expression of a fungal ferulic acid esterase increases cell wall digestibility of tall fescue (*Festuca arundinacea*). *Plant Biotechnol. J.* **6**, 264-280.

Buanafina, M.M.d.O., Langdon, T., Hauck, B., Dalton, S., Timms-Taravella, E. and Morris, P. (2010) Targeting expression of a fungal ferulic acid esterase to the apoplast, endoplasmic reticulum or golgi can disrupt feruloylation of the growing cell wall and increase the biodegradability of tall fescue (*Festuca arundinacea*). *Plant Biotechnol. J.* **8**, 316-331.

Buanafina, M.M.d.O., Langdon, T., Hauck, B., Dalton, S.J. and Morris, P. (2006) Manipulating the phenolic acid content and digestibility of Italian ryegrass (*Lolium multiflorum*) by vacuolar-targeted expression of a fungal ferulic acid esterase. In: *Twenty-Seventh Symposium on Biotechnology for Fuels and Chemicals* (McMillan, J., Adney, W., Mielenz, J. and Klasson, K.T. eds), pp. 416-426. Humana Press.

Bugg, T.D.H., Ahmad, M., Hardiman, E.M. and Rahmanpour, R. (2011) Pathways for degradation of lignin in bacteria and fungi. *Nat. Prod. Rep.* **28**, 1883-1896.

Chatterjee, A., Das, N.C., Raha, S., Babbit, R., Huang, Q.W., Zaitlin, D. and Maiti, I.B. (2010) Production of xylanase in transgenic tobacco for industrial use in bioenergy and biofuel applications. *In Vitro Cell. Dev. Biol. Plant* **46**, 198-209.

Cho, K.M., Hong, S.Y., Lee, S.M., Kim, Y.H., Kahng, G.G., Kim, H. and Yun, H.D. (2006) A cel44C-man26A gene of endophytic *Paenibacillus polymyxa* GS01 has multi-glycosyl hydrolases in two catalytic domains. *Appl. Microbiol. Biotechnol.* **73**, 618-630.

Chou, H., Dai, Z.Y., Hsieh, C. and Ku, M. (2011) High level expression of *Acidothermus cellulolyticus* β-1, 4-endoglucanase in transgenic rice enhances the hydrolysis of its straw by cultured cow gastric fluid. *Biotechnol. Biofuels* **4**, 58.

Clough, R.C., Pappu, K., Thompson, K., Beifuss, K., Lane, J., Delaney, D.E., Harkey, R., Drees, C., Howard, J.A. and Hood, E.E. (2006) Manganese peroxidase from the white-rot fungus *Phanerochaete chrysosporium* is enzymatically active and accumulates to high levels in transgenic maize seed. *Plant Biotechnol. J.* **4**, 53-62.

Cohen, S., Belinky, P.A., Hadar, Y. and Dosoretz, C.G. (2009) Characterization of catechol derivative removal by lignin peroxidase in aqueous mixture. *Bioresour. Technol.* **100**, 2247-2253.

Cordula, C.R., Lima, M.A., Shinjo, S.K., Gesteira, T.F., Pol-Fachin, L., Coulson-Thomas, V.J., Verli, H., Yates, E.A., Rudd, T.R., Pinhal, M.A.S., Toma, L., Dietrich, C.P., Nader, H.B. and Tersariol, I.L.S. (2014) On the catalytic mechanism of polysaccharide lyases: evidence of His and Tyr involvement in heparin lysis by heparinase I and the role of Ca2+. *Mol. Biosyst.* **10**, 54-64.

Culleton, H., McKie, V. and de Vries, R.P. (2013) Physiological and molecular aspects of degradation of plant polysaccharides by fungi: What have we learned from Aspergillus? *Biotechnol. J.* **8**, 884-894.

Dai, Z.Y., Hooker, B.S., Quesenberry, R.D. and Thomas, S.R. (2005) Optimization of *Acidothermus cellulolyticus* endoglucanase (E1) production in transgenic tobacco plants by transcriptional, post-transcription and post-translational modification. *Transgenic Res.* **14**, 627-643.

Devaiah, S.P., Requesens, D.V., Chang, Y.-K., Hood, K.R., Flory, A., Howard, J.A. and Hood, E.E. (2013) Heterologous expression of cellobiohydrolase II (Cel6A) in maize endosperm. *Transgenic Res.* **22**, 477-488.

Egelkrout, E., McGaughey, K., Keener, T., Ferleman, A., Woodard, S., Devaiah, S., Nikolov, Z., Hood, E. and Howard, J. (2013) Enhanced expression levels of cellulase enzymes using multiple transcription units. *Bioenerg. Res.* **6**, 699-710.

Elleuche, S. (2015) Bringing functions together with fusion enzymes—from nature's inventions to biotechnological applications. *Appl. Microbiol. Biotechnol.* **99**, 1545-1556.

Espinoza-Sánchez, E.A., Álvarez-Hernández, M.H., Torres-Castillo, J.A., Rascón-Cruz, Q., Gutiérrez-Díez, A., Zavala-García, F. and Sinagawa-García, S.R. (2015) Stable expression and characterization of a fungal pectinase and bacterial peroxidase genes in tobacco chloroplast. *Electron. J. Biotechnol.* **18**, 161-168.

Fan, Z.M. and Yuan, L. (2010) Production of multifunctional chimaeric enzymes in plants: a promising approach for degrading plant cell wall from within. *Plant Biotechnol. J.* **8**, 308-315.

Feng, C.L., Zeng, G.M., Huang, D.L., Hu, S., Zhao, M.H., Lai, C., Huang, C., Wei, Z. and Li, N.J. (2011) Effect of ligninolytic enzymes on lignin degradation and carbon utilization during lignocellulosic waste composting. *Process Biochem.* **46**, 1515-1520.

Ferrer, M., Ghazi, A., Beloqui, A., Vieites, J.M., López-Cortés, N., Marín-Navarro, J., Nechitaylo, T.Y., Guazzaroni, M.-E., Polaina, J., Waliczek, A., Chernikova, T.N., Reva, O.N., Golyshina, O.V. and Golyshin, P.N. (2012) Functional metagenomics unveils a multifunctional glycosyl hydrolase from the family 43 catalysing the breakdown of plant polymers in the calf rumen. *PLoS One* **7**, e38134.

Furukawa, K., Ichikawa, S., Nigorikawa, M., Sonoki, T. and Ito, Y. (2014) Enhanced production of reducing sugars from transgenic rice expressing exo-glucanase under the control of a senescence-inducible promoter. *Transgenic Res.* **23**, 531-537.

Furukawa, T., Sawaguchi, C., Watanabe, A., Takahashi, M., Nigorikawa, M., Furukawa, K., Iimura, Y., Kajita, S., Oguchi, T., Ito, Y. and Sonoki, T. (2013) Application of fungal laccase fused with cellulose-binding domain to develop low-lignin rice plants. *J. Biosci. Bioeng.* **116**, 616-619.

Gao, D.H., Uppugundla, N., Chundawat, S., Yu, X.R., Hermanson, S., Gowda, K., Brumm, P., Mead, D., Balan, V. and Dale, B. (2011) Hemicellulases and auxiliary enzymes for improved conversion of lignocellulosic biomass to monosaccharides. *Biotechnol. Biofuels* **4**, 5.

Garron, M.-L. and Cygler, M. (2010) Structural and mechanistic classification of uronic acid-containing polysaccharide lyases. *Glycobiology* **20**, 1547-1573.

Gibson, L.J. (2012) The hierarchical structure and mechanics of plant materials. *J. R. Soc. Interface* **9**, 2749-2766.

Gilbert, H.J. (2010) The biochemistry and structural biology of plant cell wall deconstruction. *Plant Physiol.* **153**, 444-455.

Gilbert, H.J., Knox, J.P. and Boraston, A.B. (2013) Advances in understanding the molecular basis of plant cell wall polysaccharide recognition by carbohydrate-binding modules. *Curr. Opin. Struct. Biol.* **23**, 669-677.

Gray, B.N., Ahner, B.A. and Hanson, M.R. (2009) High-level bacterial cellulase accumulation in chloroplast-transformed tobacco mediated by downstream box fusions. *Biotechnol. Bioeng.* **102**, 1045-1054.

Gray, B.N., Bougri, O., Carlson, A.R., Meissner, J., Pan, S.H., Parker, M.H., Zhang, D.C., Samoylov, V., Ekborg, N.A. and Michael Raab, R. (2011a) Global and grain-specific accumulation of glycoside hydrolase family 10 xylanases in transgenic maize (Zea mays). *Plant Biotechnol. J.* **9**, 1100-1108.

Gray, B.N., Yang, H.J., Ahner, B.A. and Hanson, M.R. (2011b) An efficient downstream box fusion allows high-level accumulation of active bacterial beta-glucosidase in tobacco chloroplasts. *Plant Mol. Biol.* **76**, 345-355.

Hägglund, P., Eriksson, T., Collén, A., Nerinckx, W., Claeyssens, M. and Stålbrand, H. (2003) A cellulose-binding module of the *Trichoderma reesei* β-mannanase Man5A increases the mannan-hydrolysis of complex substrates. *J. Biotechnol.* **101**, 37-48.

Hahn, S., Giritch, A., Bartels, D., Bortesi, L. and Gleba, Y. (2014) A novel and fully scalable *Agrobacterium* spray-based process for manufacturing cellulases and other cost-sensitive proteins in plants. *Plant Biotechnol. J.*, n/a-n/a.

Harholt, J., Bach, I.C., Lind-Bouquin, S., Nunan, K.J., Madrid, S.M., Brinch-Pedersen, H., Holm, P.B. and Scheller, H.V. (2010) Generation of transgenic wheat (*Triticum aestivum* L.) accumulating heterologous endo-xylanase or ferulic acid esterase in the endosperm. *Plant Biotechnol. J.* **8**, 351-362.

Harrison, M., Zhang, Z.Y., Shand, K., Chong, B., Nichols, J., Oeller, P., O'Hara, I., Doherty, W. and Dale, J. (2014a) The combination of plant-expressed cellobiohydrolase and low dosages of cellulases for the hydrolysis of sugar cane bagasse. *Biotechnol. Biofuels* **7**, 131.

Harrison, M.D., Geijskes, J., Coleman, H.D., Shand, K., Kinkema, M., Palupe, A., Hassall, R., Sainz, M., Lloyd, R., Miles, S. and Dale, J.L. (2011) Accumulation of recombinant cellobiohydrolase and endoglucanase in the leaves of mature transgenic sugar cane. *Plant Biotechnol. J.* **9**, 884-896.

Harrison, M.D., Geijskes, R.J., Lloyd, R., Miles, S., Palupe, A., Sainz, M.B. and Dale, J.L. (2014b) Recombinant cellulase accumulation in the leaves of mature, vegetatively propagated transgenic sugarcane. *Mol. Biotechnol.* **56**, 795-802.

Hatfield, R.D., Ralph, J. and Grabber, J.H. (1999) Cell wall cross-linking by ferulates and diferulates in grasses. *J. Sci. Food Agric.* **79**, 403-407.

Hofrichter, M. (2002) Review: lignin conversion by manganese peroxidase (MnP). *Enzyme Microb. Technol.* **30**, 454-466.

Hood, E.E., Devaiah, S.P., Fake, G., Egelkrout, E., Teoh, K.T., Requesens, D.V., Hayden, C., Hood, K.R., Pappu, K.M., Carroll, J. and Howard, J.A. (2012) Manipulating corn germplasm to increase recombinant protein accumulation. *Plant Biotechnol. J.* **10**, 20-30.

Hood, E.E., Love, R., Lane, J., Bray, J., Clough, R., Pappu, K., Drees, C., Hood, K.R., Yoon, S., Ahmad, A. and Howard, J.A. (2007) Subcellular targeting is a key condition for high-level accumulation of cellulase protein in transgenic maize seed. *Plant Biotechnol. J.* **5**, 709-719.

Hood, N.C., Hood, K.R., Woodard, S.L., Devaiah, S.P., Jeoh, T., Wilken, L., Nikolov, Z., Egelkrout, E., Howard, J.A. and Hood, E.E. (2014) Purification and characterization of recombinant Cel7A from maize seed. *Appl. Biochem. Biotechnol.* **174**, 2864-2874.

Hoshikawa, K., Endo, S., Mizuniwa, S., Makabe, S., Takahashi, H. and Nakamura, I. (2012) Transgenic tobacco plants expressing endo-β-mannanase gene from deep-sea *Bacillus* sp. JAMB-602 strain confer enhanced resistance against fungal pathogen (*Fusarium oxysporum*). *Plant Biotechnol Rep* **6**, 243-250.

Hurlbert, J.C. and Preston, J.F., III (2001) Functional characterization of a novel xylanase from a corn strain of *Erwinia chrysanthemi*. *J. Bacteriol.* **183**, 2093-2100.

Hwang, M., Lindenmuth, B., McDonald, K. and Falk, B. (2012) Bipartite and tripartite Cucumber mosaic virus-based vectors for producing the Acidothermus cellulolyticus endo-1,4-beta-glucanase and other proteins in non-transgenic plants. *BMC Biotechnol.* **12**, 66.

Jabbour, D., Angelos, E., Mukhopadhyay, A., Womboldt, A., Borrusch, M. and Walton, J. (2014) Factors contributing to the recalcitrance of herbaceous dicotyledons (forbs) to enzymatic deconstruction. *Biotechnol. Biofuels* **7**, 52.

Janusz, G., Kucharzyk, K.H., Pawlik, A., Staszczak, M. and Paszczynski, A.J. (2013) Fungal laccase, manganese peroxidase and lignin peroxidase: Gene expression and regulation. *Enzyme Microb. Technol.* **52**, 1-12.

Jiang, X.-R., Zhou, X.-Y., Jiang, W.-Y., Gao, X.-R. and Li, W.-L. (2011) Expressions of thermostable bacterial cellulases in tobacco plant. *Biotechnol. Lett.* **33**, 1797-1803.

Jin, S.X., Kanagaraj, A., Verma, D., Lange, T. and Daniell, H. (2011) Release of hormones from conjugates: Chloroplast expression of β-Glucosidase results in elevated phytohormone levels associated with significant increase in biomass and protection from aphids or whiteflies conferred by sucrose esters. *Plant Physiol.* **155**, 222-235.

Jung, S., Kim, S., Bae, H., Lim, H.-S. and Bae, H.-J. (2010) Expression of thermostable bacterial beta-glucosidase (BglB) in transgenic tobacco plants. *Bioresour. Technol.* **101**, 7144-7150.

Jung, S., Lee, D.-S., Kim, Y.-O., Joshi, C.P. and Bae, H.-J. (2013) Improved recombinant cellulase expression in chloroplast of tobacco through promoter engineering and 5' amplification promoting sequence. *Plant Mol. Biol.* **83**, 317-328.

Jung, S.-K., Lindenmuth, B.E., McDonald, K.A., Hwang, M.S., Bui, M.Q.N., Falk, B.W., Uratsu, S.L., Phu, M.L. and Dandekar, A.M. (2014) Agrobacterium tumefaciens mediated transient expression of plant cell wall-degrading enzymes in detached sunflower leaves. *Biotechnol. Prog.* **30**, 905-915.

Kim, J.Y., Kavas, M., Fouad, W.M., Nong, G., Preston, J.F. and Altpeter, F. (2011) Production of hyperthermostable GH10 xylanase Xyl10B from Thermotoga maritima in transplastomic plants enables complete hydrolysis of methylglucuronoxylan to fermentable sugars for biofuel production. *Plant Mol. Biol.* **76**, 357-369.

Kim, S., Lee, D.-S., Choi, I.S., Ahn, S.-J., Kim, Y.-H. and Bae, H.-J. (2010) *Arabidopsis thaliana* Rubisco small subunit transit peptide increases the accumulation of *Thermotoga maritima* endoglucanase Cel5A in chloroplasts of transgenic tobacco plants. *Transgenic Res.* **19**, 489-497.

Kimura, T., Mizutani, T., Sun, J.-L., Kawazu, T., Karita, S., Sakka, M., Kobayashi, Y., Ohmiya, K. and Sakka, K. (2010) Stable production of thermotolerant xylanase B of *Clostridium stercorarium* in transgenic tobacco and rice. *Biosci. Biotechnol. Biochem.* **74**, 954-960.

Klose, H., Günl, M., Usadel, B., Fischer, R. and Commandeur, U. (2013) Ethanol inducible expression of a mesophilic cellulase avoids adverse effects on plant development. *Biotechnol. Biofuels* **6**, 53.

Klose, H., Günl, M., Usadel, B., Fischer, R. and Commandeur, U. (2015) Cell wall modification in tobacco by differential targeting of recombinant endoglucanase from *Trichoderma reesei*. *BMC Plant Biol.*, 54.

Klose, H., Röder, J., Girfoglio, M., Fischer, R. and Commandeur, U. (2012) Hyperthermophilic endoglucanase for *in planta* lignocellulose conversion. *Biotechnol. Biofuels* **5**, 63.

Kolotilin, I., Kaldis, A., Pereira, E.O., Laberge, S. and Menassa, R. (2013) Optimization of transplastomic production of hemicellulases in tobacco: effects of expression cassette configuration and tobacco cultivar used as production platform on recombinant protein yields. *Biotechnol. Biofuels* **6**, 65.

Koseki, T., Fushinobu, S., Ardiansyah, Shirakawa, H. and Komai, M. (2009) Occurrence, properties, and applications of feruloyl esterases. *Appl. Microbiol. Biotechnol.* **84**, 803-810.

Latha Gandla, M., Derba-Maceluch, M., Liu, X.K., Gerber, L., Master, E.R., Mellerowicz, E.J. and Jönsson, L.J. (2015) Expression of a fungal glucuronoyl esterase in Populus: Effects on wood properties and saccharification efficiency. *Phytochemistry* **112**, 210-220.

Lee, D.-S., Lee, K.-H., Jung, S., Jo, E.-J., Han, K.-H. and Bae, H.-J. (2012) Synergistic effects of 2A-mediated polyproteins on the production of lignocellulose degradation enzymes in tobacco plants. *J. Exp. Bot.* **63**, 4797-4810.

Levasseur, A., Drula, E., Lombard, V., Coutinho, P.M. and Henrissat, B. (2013) Expansion of the enzymatic repertoire of the CAZy database to integrate auxiliary redox enzymes. *Biotechnol. Biofuels* **6**, 41.

Liers, C., Arnstadt, T., Ullrich, R. and Hofrichter, M. (2011) Patterns of lignin degradation and oxidative enzyme secretion by different wood- and litter-colonizing basidiomycetes and ascomycetes grown on beech-wood. *FEMS Microbiol. Ecol.* **78**, 91-102.

Llop-Tous, I., Ortiz, M., Torrent, M. and Ludevid, M.D. (2011) The expression of a xylanase targeted to ER-protein bodies provides a simple strategy to produce active insoluble enzyme polymers in tobacco plants. *PLoS One* **6**.

Lombard, V., Bernard, T., Rancurel, C., Brumer, H., Coutinho, P.M. and Henrissat, B. (2010) A hierarchical classification of polysaccharide lyases for glycogenomics. *Biochem. J.* **432**, 437-444.

Lombard, V., Golaconda Ramulu, H., Drula, E., Coutinho, P.M. and Henrissat, B. (2014) The carbohydrate-active enzymes database (CAZy) in 2013. *Nucleic Acids Res.* **42**, D490-495.

Lundell, T.K., Mäkelä, M.R. and Hildén, K. (2010) Lignin-modifying enzymes in filamentous basidiomycetes--ecological, functional and phylogenetic review. *J. Basic Microbiol.* **50**, 5-20.

Mahadevan, S.A., Wi, S.G., Kim, Y.O., Lee, K.H. and Bae, H.-J. (2011) In planta differential targeting analysis of Thermotoga maritima Cel5A and CBM6-engineered Cel5A for autohydrolysis. *Transgenic Res.* **20**, 877-886.

Mei, C.S., Park, S.-H., Sabzikar, R., Ransom, C., Qi, C.F. and Sticklen, M. (2009) Green tissue-specific production of a microbial endo-cellulase in maize (*Zea mays* L.) endoplasmic-reticulum and mitochondria converts cellulose into fermentable sugars. *J. Chem. Technol. Biotechnol.* **84**, 689-695.

Mohnen, D. (2008) Pectin structure and biosynthesis. *Curr. Opin. Plant Biol.* **11**, 266-277.

Nakahira, Y., Ishikawa, K., Tanaka, K., Tozawa, Y. and Shiina, T. (2013) Overproduction of hyperthermostable β-1,4-endoglucanase from the archaeon *Pyrococcus horikoshii* by tobacco chloroplast engineering. *Biosci. Biotechnol. Biochem.* **77**, 2140-2143.

Nigorikawa, M., Watanabe, A., Furukawa, K., Sonoki, T. and Ito, Y. (2012) Enhanced saccharification of rice straw by overexpression of rice exo-glucanase. *Rice* **5**, 14.

Nutt, A., Sild, V., Pettersson, G. and Johansson, G. (1998) Progress curves: A mean for functional classification of cellulases. *Eur. J. Biochem.* **258**, 200-206.
Oraby, H., Venkatesh, B., Dale, B., Ahmad, R., Ransom, C., Oehmke, J. and Sticklen, M. (2007) Enhanced conversion of plant biomass into glucose using transgenic rice-produced endoglucanase for cellulosic ethanol. *Transgenic Res.* **16**, 739-749.
Pantaleoni, L., Longoni, P., Ferroni, L., Baldisserotto, C., Leelavathi, S., Reddy, V.S., Pancaldi, S. and Cella, R. (2014) Chloroplast molecular farming: efficient production of a thermostable xylanase by Nicotiana tabacum plants and long-term conservation of the recombinant enzyme. *Protoplasma* **251**, 639-648.
Park, S.-H., Ransom, C., Mei, C.S., Sabzikar, R., Qi, C.F., Chundawat, S., Dale, B. and Sticklen, M. (2011) The quest for alternatives to microbial cellulase mix production: corn stover-produced heterologous multi-cellulases readily deconstruct lignocellulosic biomass into fermentable sugars. *J. Chem. Technol. Biotechnol.* **86**, 633-641.
Pawar, P.M.-A., Koutaniemi, S., Tenkanen, M. and Mellerowicz, E.J. (2013) Acetylation of woody lignocellulose: significance and regulation. *Front. Plant Sci.* **4**.
Pereira, E., Kolotilin, I., Conley, A. and Menassa, R. (2014) Production and characterization of in planta transiently produced polygalacturanase from Aspergillus niger and its fusions with hydrophobin or ELP tags. *BMC Biotechnol.* **14**, 59.
Petersen, K. and Bock, R. (2011) High-level expression of a suite of thermostable cell wall-degrading enzymes from the chloroplast genome. *Plant Mol. Biol.* **76**, 311-321.
Pogorelko, G., Fursova, O., Lin, M., Pyle, E., Jass, J. and Zabotina, O.A. (2011) Post-synthetic modification of plant cell walls by expression of microbial hydrolases in the apoplast. *Plant Mol. Biol.* **77**, 433-445.
Pogorelko, G., Lionetti, V., Fursova, O., Sundaram, R.M., Qi, M., Whitham, S.A., Bogdanove, A.J., Bellincampi, D. and Zabotina, O.A. (2013) Arabidopsis and *Brachypodium distachyon* transgenic plants expressing *Aspergillus nidulans* acetylesterases have decreased degree of polysaccharide acetylation and increased resistance to pathogens. *Plant Physiol.* **162**, 9-23.
Ransom, C., Balan, V., Biswas, G., Dale, B., Crockett, E. and Sticklen, M. (2007) Heterologous *Acidothermus cellulolyticus* 1,4-β-endoglucanase E1 produced within the corn biomass converts corn stover into glucose. *Appl. Biochem. Biotechnol.* **137-140**, 207-219.
Scheller, H.V. and Ulvskov, P. (2010) Hemicelluloses. *Ann. Rev. Plant Biol.* **61**, 263-289.
Shallom, D. and Shoham, Y. (2003) Microbial hemicellulases. *Curr. Opin. Microbiol.* **6**, 219-228.
Shen, B.X., Sun, X.G., Zuo, X., Shilling, T., Apgar, J., Ross, M., Bougri, O., Samoylov, V., Parker, M., Hancock, E., Lucero, H., Gray, B., Ekborg, N.A., Zhang, D.C., Johnson, J.C.S., Lazar, G. and Raab, R.M. (2012) Engineering a thermoregulated intein-modified xylanase into maize for consolidated lignocellulosic biomass processing. *Nat. Biotechnol.* **30**, 1131-1136.
Sun, Y., Cheng, J.J., Himmel, M.E., Skory, C.D., Adney, W.S., Thomas, S.R., Tisserat, B., Nishimura, Y. and Yamamoto, Y.T. (2007) Expression and characterization of *Acidothermus cellulolyticus* E1 endoglucanase in transgenic duckweed Lemna minor 8627. *Bioresour. Technol.* **98**, 2866-2872.

Tomassetti, S., Pontiggia, D., Verrascina, I., Reca, I.B., Francocci, F., Salvi, G., Cervone, F. and Ferrari, S. (2015) Controlled expression of pectic enzymes in *Arabidopsis thaliana* enhances biomass conversion without adverse effects on growth. *Phytochemistry* **112**, 221-230.

Tsai, A.Y.L., Canam, T., Gorzsás, A., Mellerowicz, E.J., Campbell, M.M. and Master, E.R. (2012) Constitutive expression of a fungal glucuronoyl esterase in Arabidopsis reveals altered cell wall composition and structure. *Plant Biotechnol. J.* **10**, 1077-1087.

van den Brink, J. and de Vries, R.P. (2011) Fungal enzyme sets for plant polysaccharide degradation. *Appl. Microbiol. Biotechnol.* **91**, 1477-1492.

Verma, D., Jin, S.X., Kanagaraj, A., Singh, N.D., Daniel, J., Kolattukudy, P.E., Miller, M. and Daniell, H. (2013) Expression of fungal cutinase and swollenin in tobacco chloroplasts reveals novel enzyme functions and/or substrates. *PLoS One* **8**, e57187.

Verma, D., Kanagaraj, A., Jin, S.X., Singh, N.D., Kolattukudy, P.E. and Daniell, H. (2010) Chloroplast-derived enzyme cocktails hydrolyse lignocellulosic biomass and release fermentable sugars. *Plant Biotechnol. J.* **8**, 332-350.

Weng, X.Y., Huang, Y.Y., Hou, C.X. and Jiang, D. (2013) Effects of an exogenous xylanase gene expression on the growth of transgenic rice and the expression level of endogenous xylanase inhibitor gene *RIXI*. *J. Sci. Food Agric.* **93**, 173-179.

Wong, D.W.S. (2009) Structure and action mechanism of ligninolytic enzymes. *Appl. Biochem. Biotechnol.* **157**, 174-209.

Xu, X.L., Zhang, Y.H., Meng, Q.C., Meng, K., Zhang, W., Zhou, X.J., Luo, H.Y., Chen, R.M., Yang, P.L. and Yao, B. (2013) Overexpression of a fungal β-mannanase from *Bispora* sp. MEY-1 in maize seeds and enzyme characterization. *PLoS One* **8**, e56146.

Yang, P.L., Wang, Y.R., Bai, Y.G., Meng, K., Luo, H.Y., Yuan, T.Z., Fan, Y.L. and Yao, B. (2007) Expression of xylanase with high specific activity from *Streptomyces olivaceoviridis* A1 in transgenic potato plants (*Solanum tuberosum* L.). *Biotechnol. Lett.* **29**, 659-667.

Yang, W.X., Zhang, Y.H., Zhou, X.J., Zhang, W., Xu, X.L., Chen, R.M., Meng, Q.C., Yuan, J.H., Yang, P.L. and Yao, B. (2015) Production of a highly protease-resistant fungal α-galactosidase in transgenic maize seeds for simplified feed processing. *PLoS One* **10**, e0129294.

Yu, L.-X., Gray, B.N., Rutzke, C.J., Walker, L.P., Wilson, D.B. and Hanson, M.R. (2007) Expression of thermostable microbial cellulases in the chloroplasts of nicotine-free tobacco. *J. Biotechnol.* **131**, 362-369.

Zhang, Q., Zhang, W., Lin, C.Y., Xu, X.L. and Shen, Z.C. (2012) Expression of an *Acidothermus cellulolyticus* endoglucanase in transgenic rice seeds. *Protein Expr. Purif.* **82**, 279-283.

Zhang, Y.H., Xu, X.L., Zhou, X.J., Chen, R.M., Yang, P.L., Meng, Q.C., Meng, K., Luo, H.Y., Yuan, J.H., Yao, B. and Zhang, W. (2013) Overexpression of an acidic endo-β-1,3-1,4-glucanase in transgenic maize seed for direct utilization in animal feed. *PLoS One* **8**, e81993.

Ziegelhoffer, T., Raasch, J.A. and Austin-Phillips, S. (2009) Expression of *Acidothermus cellulolyticus* E1 endo-beta-1,4-glucanase catalytic domain in transplastomic tobacco. *Plant Biotechnol. J.* **7**, 527-536.
